# Supplementary material for: A Model of the Effect of Uncertainty on the C elegans L2/L2d Decision
Source: PLoS One. 2014 Jul 16;9(7):e100580. doi: 10.1371/journal.pone.0100580 (PMC4100763; doi:10.1371/journal.pone.0100580)

Setup

(Local) In[1]:=
```
{NotebookFileName[], DateString[]}
```

(Local) Out[1]=
```
{/Volumes/leon Home/papers/return/calcs/calculations_v2.nb, Mon 3 Mar 2014 13:39:39}
```

(Local) In[2]:=
```
Get[FileNameJoin[{FileNameDrop[NotebookFileName[]], "init_v3.m"}]]
```

(Local) In[3]:=
```
rWT
```

(Local) Out[3]=
```
{broodSize → 327, cutterSpermRate → 23.6, dauerDelay → 20.9255, dauerFormation → 15.8745,
 dauerRecovery → 14.5216, dauerStage → 30.3961, dauerValue → 1., delay → 3.68301,
 hatchTime → 17.6784, l1Molt → 32.2, l1Stage → 14.5216, l1Value → 0.219292,
 l2dDelay → 7.57647, l2dStage → 16.4157, l2Molt → 41.0392, l2Stage → 8.83922,
 l2Value → 0.852381, l3Molt → 50.5098, l3Stage → 9.47059, l3Value → 1.23734,
 l4Molt → 62.5059, l4Stage → 11.9961, l4Value → 1.84461, layTime → 3.78824,
 minL2dDelay → 3.15686, minL2dStage → 11.9961, newDauerValue → 0.512063, progenyRate → 5.3,
 progenyStart → 64.4, spermRate → 66.1538, yaValue → 3.05888, λcutter → 0.0274909,
 λgrowth → 0.0637344, λhat → 0.0421625, λmax → 0.0675087, λt3 → 0.0655343}
```

Developmental timing, parameter estimation

Population growth

From Hodgkin and Barnes: At 20C, an egg takes 64.4 hours to mature to the point at which it produces its first egg. Thereafter it produces 5.3/hour until the sperm supply runs out. It has 327 sperm.

(Local) In[4]:=
```
prWT = {progenyStart → 64.4, progenyRate → 5.3, broodSize → 327}
```

(Local) Out[4]=
```
{progenyStart → 64.4, progenyRate → 5.3, broodSize → 327}
```

(Local) In[5]:=
```
rWT = prWT
```

(Local) Out[5]=
```
{progenyStart → 64.4, progenyRate → 5.3, broodSize → 327}
```

Present value in eggs of an egg at time 0 with discount rate λ:

(Local) In[6]:=
```
Clear[pv1];
pv1[λ_, progenyStart_: 64.4, progenyRate_: 5.3, broodSize_: 327] :=
  Evaluate[
   ∫_progenyStart^(progenyStart+broodSize/progenyRate) ⅇ^(-λ t) progenyRate ⅆt
  ];
pv1[0, progenyStart_: 64.4, progenyRate_: 5.3, broodSize_: 327] :=
  Evaluate[
   ∫_progenyStart^(progenyStart+broodSize/progenyRate) (ⅇ^(-λ t) progenyRate /. λ → 0) ⅆt
  ];
{pv1[λ], pv1[0]}
```

(Local) Out[9]=

$$\left\{\frac{5.3\, e^{-126.098\,\lambda}\left(-1+e^{61.6981\,\lambda}\right)}{\lambda},\ 327\right\}$$

Internal rate of return:

(Local) In[10]:=
```
FindRoot[1 == pv1[λmax], {λmax, 0.05}]
```

(Local) Out[10]=
```
{λmax → 0.0675087}
```

(Local) In[11]:=
```
rWT = Union[rWT, FindRoot[1 == pv1[λmax], {λmax, 0.05}]]
```

(Local) Out[11]=
```
{broodSize → 327, progenyRate → 5.3, progenyStart → 64.4, λmax → 0.0675087}
```

(Local) In[12]:=
```
Log[2] / λmax /. rWT
```

(Local) Out[12]=
```
10.2675
```

Event times

I culled the following times from the literature (see papers\return\Book1.xls) and Wood, fig 6, in the 1988 Worm book. He gives the life cycle, fertilization to fertilization, as 51h at 25C. To correct his numbers for temperature:

(Local) In[13]:=
```
c25to20 = progenyStart / 51 /. rWT
```

(Local) Out[13]=
```
1.26275
```

These are the times of other events in his diagram:

(Local) In[14]:=
```
w25times = {layTime → 3, hatchTime → 14,
   l1Molt → 255 / 10, l2Molt → 325 / 10, l3Molt → 40, l4Molt → 495 / 10}
```

(Local) Out[14]=

$$\left\{\text{layTime} \to 3,\ \text{hatchTime} \to 14,\ \text{l1Molt} \to \frac{51}{2},\ \text{l2Molt} \to \frac{65}{2},\ \text{l3Molt} \to 40,\ \text{l4Molt} \to \frac{99}{2}\right\}$$

(Local) In[15]:=
```
wtTimes = (#[[1]] -> c25to20 * #[[2]]) & /@ w25times
```

(Local) Out[15]=
```
{layTime → 3.78824, hatchTime → 17.6784, l1Molt → 32.2,
 l2Molt → 41.0392, l3Molt → 50.5098, l4Molt → 62.5059}
```

(Local) In[16]:=
```
rWT = Union[rWT, wtTimes]
```

(Local) Out[16]=
```
{broodSize → 327, hatchTime → 17.6784, l1Molt → 32.2,
 l2Molt → 41.0392, l3Molt → 50.5098, l4Molt → 62.5059, layTime → 3.78824,
 progenyRate → 5.3, progenyStart → 64.4, λmax → 0.0675087}
```

L2d

A dauer larva, according to Riddle in the 88 Worm book, takes 10-13 hours to reach the molt at 25C, under which conditions he shows the life cycle as 8 + 12 + 7 + 7 + 9 + 8 hours.

(Local) In[17]:=
```
{cRiddleto20 = (progenyStart /. rWT) / (8 + 12 + 7 + 7 + 9 + 8), c25to20}
```

(Local) Out[17]=
```
{1.26275, 1.26275}
```

That's reassuring.

According to Golden and Riddle, the L2d stage lasts 6h longer than L2 at 25C. Under minimally inducing conditions the delay is 2-3h.

(Local) In[18]:=
```
l2dr1 = {l2dDelay → 6 cRiddleto20, minL2dDelay → 2.5 cRiddleto20}
```

(Local) Out[18]=
```
{l2dDelay → 7.57647, minL2dDelay → 3.15686}
```

(Local) In[19]:=
```
rWT = Union[rWT, l2dr1]
```

(Local) Out[19]=
```
{broodSize → 327, hatchTime → 17.6784, l1Molt → 32.2, l2dDelay → 7.57647,
 l2Molt → 41.0392, l3Molt → 50.5098, l4Molt → 62.5059, layTime → 3.78824,
 minL2dDelay → 3.15686, progenyRate → 5.3, progenyStart → 64.4, λmax → 0.0675087}
```

Stage durations

(Local) In[20]:=
```
rWT = rWT ~ Union ~
   {l1Stage → l1Molt - hatchTime /. rWT,
    l2Stage → l2Molt - l1Molt /. rWT,
    l2dStage → l2Molt - l1Molt + l2dDelay /. rWT,
    minL2dStage → l2Molt - l1Molt + minL2dDelay /. rWT,
    l3Stage → l3Molt - l2Molt /. rWT,
    l4Stage → l4Molt - l3Molt /. rWT}
```

(Local) Out[20]=
```
{broodSize → 327, hatchTime → 17.6784, l1Molt → 32.2, l1Stage → 14.5216,
 l2dDelay → 7.57647, l2dStage → 16.4157, l2Molt → 41.0392, l2Stage → 8.83922,
 l3Molt → 50.5098, l3Stage → 9.47059, l4Molt → 62.5059, l4Stage → 11.9961,
 layTime → 3.78824, minL2dDelay → 3.15686, minL2dStage → 11.9961,
 progenyRate → 5.3, progenyStart → 64.4, λmax → 0.0675087}
```

Growth rate and α

This is the Laplace transform of the time to reach m for a Brownian motion with drift $\mu < 0$.

$$\mathbb{E}e^{-u\tau_m} = e^{m\mu - m\sqrt{2u+\mu^2}}$$

(Local) In[21]:= `lt1 = e^(m μ - m Sqrt[2 u + μ^2])`

(Local) Out[21]= $e^{m\mu - m\sqrt{2u+\mu^2}}$

(Local) In[22]:= `Assuming[μ < 0, Simplify[lt1 /. u → 0]]`

(Local) Out[22]= $e^{2m\mu}$

$\frac{a(t)}{\delta\nu}$ is a Brownian motion with drift $\mu = -\frac{\alpha}{\nu}$. The Laplace transform of the time to reach a is

(Local) In[23]:= `lt2 = (lt1 /. {m → a/(δ ν), μ → -α/ν} // FullSimplify)`

(Local) Out[23]= $e^{-\frac{a\left(\alpha+\sqrt{\alpha^2+2u\nu^2}\right)}{\delta\nu^2}}$

(Local) In[24]:= `lt2 = (lt1 /. {m → a/(δ ν), μ → -α/ν} /. ν → Sqrt[2 α/λ] // FullSimplify)`

(Local) Out[24]= $e^{-\frac{a\left(\lambda+\sqrt{\lambda\left(\frac{4u}{\alpha}+\lambda\right)}\right)}{2\delta}}$

(Local) In[25]:= `lt2 /. u → 0 // Simplify`

(Local) Out[25]= $e^{-\frac{a\lambda}{\delta}}$

The PDF of the time to reach a is

(Local) In[26]:= `pdf1 = Simplify[InverseLaplaceTransform[lt2, u, τ], a > 0]`

(Local) Out[26]= $\frac{a\, e^{-\frac{\lambda(a+\alpha\delta\tau)^2}{4\alpha\delta^2\tau}}}{2\sqrt{\pi}\,\delta\sqrt{\frac{\alpha\tau^3}{\lambda}}}$

Now, I want to pick α so that the worm takes twice as long to reach the L2d molt in the wild as it does in the lab, i.e. 33 hours instead of 16. But since there is a distribution of times, it's not obvious which one should be double the lab time. My first thought was to use the mean, which is simple: $\mu_\tau = \frac{a}{\alpha}$. In other words, to double the time, I use $\alpha = 0.5$.

(Local) In[27]:= **Assuming**$\left[a > 0, \left\{\int_0^\infty \text{pdf1}\, d\tau, \int_0^\infty \tau\, \text{pdf1}\, d\tau, \frac{\int_0^\infty \tau\, \text{pdf1}\, d\tau}{\int_0^\infty \text{pdf1}\, d\tau}, \frac{\int_0^\infty \tau\, \text{pdf1}\, d\tau}{\int_0^\infty \text{pdf1}\, d\tau}\ /.\ \alpha \to 0.5\ /.\ \text{params}\right\}\right]$

(Local) Out[27]= $\left\{e^{-\frac{a\lambda}{\delta}}, \frac{a\, e^{-\frac{a\lambda}{\delta}}}{\alpha\,\delta}, \frac{a}{\alpha\,\delta}, 32.8314\right\}$

(Local) In[28]:= **Plot[Evaluate[pdf1 /. α → 0.5 /. params], {τ, 0, 30}, PlotRange → Full]**

(Local) Out[28]=

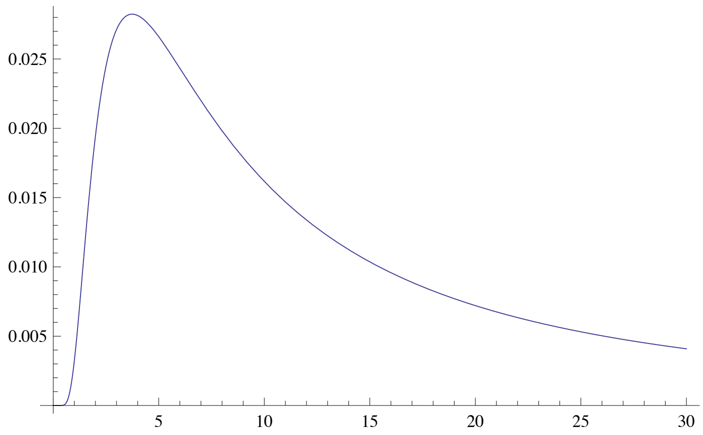

(Local) In[29]:= **Solve**$[\partial_\tau \text{pdf1} == 0, \tau]\ /.\ \alpha \to 0.5\ /.\ \text{params}$

Solve::ifun : Inverse functions are being used by Solve, so
some solutions may not be found; use Reduce for complete solution information. ≫

(Local) Out[29]= {{τ → -288.351}, {τ → 3.73814}}

(Local) In[30]:= **medianτ = τm /. FindRoot**$\left[\int_0^{\tau m} \text{pdf1}\, d\tau == 0.5\, e^{-\frac{a\lambda}{\delta}}\ /.\ \alpha \to 0.5\ /.\ \text{params}, \{\tau m, 5\}\right]$

(Local) Out[30]= 13.9439

OK, here's the problem. The mean is 33 hours, but the mode is only 3.7, median 14. It's a long-tailed distribution and the mean is not representative. One effect is that I need high volatilities to get substantial option values—the volatility only has 4 hours in which to act. Of course, this is absurd. An L2d does not develop in 3.7 h. I need to change my default α to something much slower. So instead let's try finding α such that the mode is 33 h.

(Local) In[31]:= **params2 = DeleteCases[params, α → _]**

(Local) Out[31]= {a → 8.83922, λ → 0.0421625, δ → 0.538462, dValue → 0.512063}

(Local) In[32]:= **modeτ = τ /. Solve**$[\partial_\tau \text{pdf1} == 0, \tau]$**[[2]] // Simplify**

Solve::ifun : Inverse functions are being used by Solve, so
some solutions may not be found; use Reduce for complete solution information. ≫

(Local) Out[32]= $\frac{-3\,\delta + \sqrt{9\,\delta^2 + a^2\,\lambda^2}}{\alpha\,\delta\,\lambda}$

(Local) In[33]:= **Solve**$\left[\frac{2\,a}{\delta} == \text{mode}\tau, \alpha\right]$**[[1]]**

(Local) Out[33]= $\left\{\alpha \to \frac{-3\,\delta + \sqrt{9\,\delta^2 + a^2\,\lambda^2}}{2\,a\,\lambda}\right\}$

(Local) In[34]:= $\text{Solve}\left[\frac{2\,a}{\delta} == \text{mode}\tau,\ \alpha\right][[1]]$ /. params2

(Local) Out[34]= {α → 0.0569294}

(Local) In[35]:= $\frac{\text{mode}\tau}{\frac{2\,a}{\delta}}\,\alpha$ /. params2

(Local) Out[35]= 0.0569294

Good. It's simply inversely proportional. And if I wanted to get the median to 33, I would choose

(Local) In[36]:= $\frac{\text{median}\tau}{\frac{2\,a}{\delta}}\,\alpha$ /. α → 0.5 /. params2

(Local) Out[36]= 0.212356

(Local) In[37]:=
```
Plot[Evaluate[pdf1 /. params2 /. α → 0.057], {τ, 0, 200}, PlotRange → Full,
 Epilog → {Line[2 {{m`l2Stage + m`l2dDelay, 0}, {m`l2Stage + m`l2dDelay, 1}}],
   Line[{{m`l2Stage, 0}, {m`l2Stage, 1}}],
   Line[{{m`l2Stage + m`l2dDelay, 0}, {m`l2Stage + m`l2dDelay, 1}}]}]
```

(Local) Out[37]=

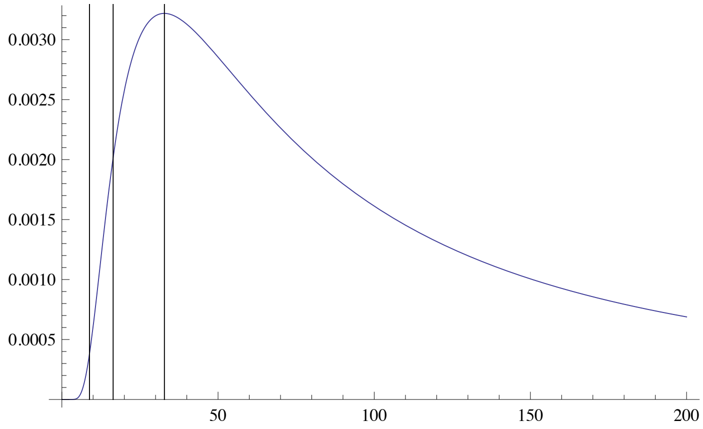

(Local) In[38]:=
```
Plot[Evaluate[pdf1 /. params2 /. α → 0.212], {τ, 0, 50}, PlotRange → Full,
 Epilog → {Line[2 {{m`l2Stage + m`l2dDelay, 0}, {m`l2Stage + m`l2dDelay, 1}}],
   Line[{{m`l2Stage, 0}, {m`l2Stage, 1}}],
   Line[{{m`l2Stage + m`l2dDelay, 0}, {m`l2Stage + m`l2dDelay, 1}}]}]
```

(Local) Out[38]=

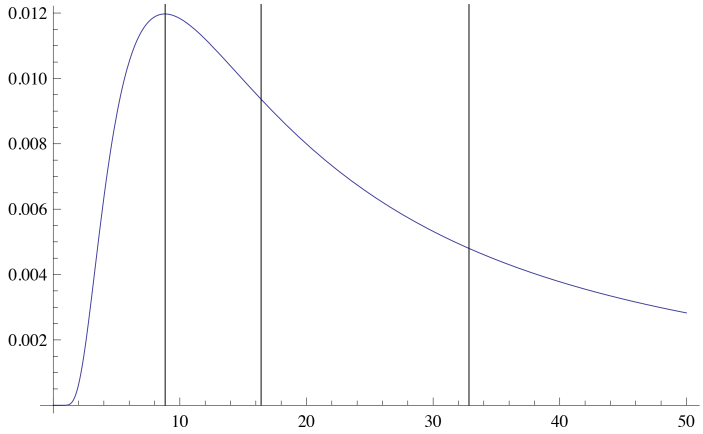

The $\alpha = 0.056$ plot looks most like what I want to see, so let's use that in future.

■ Delay of early exercise

(Local) In[39]:=
```
Solve[delay/m`δ + (m`l2Stage - delay) == m`minL2dStage, delay][[1]]
```

(Local) Out[39]=
```
{delay → 3.68301}
```

(Local) In[40]:=
```
rWT = Union[rWT, Solve[delay/m`δ + (m`l2Stage - delay) == m`minL2dStage, delay][[1]]]
```

(Local) Out[40]=
```
{broodSize → 327, delay → 3.68301, hatchTime → 17.6784,
 l1Molt → 32.2, l1Stage → 14.5216, l2dDelay → 7.57647, l2dStage → 16.4157,
 l2Molt → 41.0392, l2Stage → 8.83922, l3Molt → 50.5098, l3Stage → 9.47059,
 l4Molt → 62.5059, l4Stage → 11.9961, layTime → 3.78824, minL2dDelay → 3.15686,
 minL2dStage → 11.9961, progenyRate → 5.3, progenyStart → 64.4, λmax → 0.0675087}
```

(Local) In[41]:=
```
m`delay = delay /. Solve[delay/m`δ + (m`l2Stage - delay) == m`minL2dStage, delay][[1]]
```

(Local) Out[41]=
```
3.68301
```

(Local) In[42]:=
```
{m`delay/m`δ, m`delay, m`delay/m`δ - m`delay, m`minL2dDelay}
```

(Local) Out[42]=
```
{6.83987, 3.68301, 3.15686, 3.15686}
```

■ Dauers

(Local) In[43]:=
```
dr1 = {dauerRecovery → Mean[{10, 13}] cRiddleto20}
```

(Local) Out[43]=
```
{dauerRecovery → 14.5216}
```

(Local) In[44]:=
```
rWT = Union[rWT, dr1]
```

(Local) Out[44]=
```
{broodSize → 327, dauerRecovery → 14.5216, delay → 3.68301, hatchTime → 17.6784,
 l1Molt → 32.2, l1Stage → 14.5216, l2dDelay → 7.57647, l2dStage → 16.4157,
 l2Molt → 41.0392, l2Stage → 8.83922, l3Molt → 50.5098, l3Stage → 9.47059,
 l4Molt → 62.5059, l4Stage → 11.9961, layTime → 3.78824, minL2dDelay → 3.15686,
 minL2dStage → 11.9961, progenyRate → 5.3, progenyStart → 64.4, λmax → 0.0675087}
```

Golden and Riddle say that ten hours after the L2d molt the worms shrink radially, and an hour after that "have acquired all of the characteristics of dauer larvae including resistance to treatment with 1% SDS". This is at 25.4°C, under conditions where their measured molt times (starting from "synchronized L1 larvae") are 11, 18, 24, and 32h.

(Local) In[45]:=
```
cgr25to20 = (l4Molt - l1Molt)/(32 - 11) /. rWT
```

(Local) Out[45]=
```
1.44314
```

(Local) In[46]:=
```
df1 = {dauerFormation → (10 + 1) cgr25to20}
```

(Local) Out[46]=
```
{dauerFormation → 15.8745}
```

(Local) In[47]:=
```
rWT = Union[rWT, df1]
```

(Local) Out[47]=
```
{broodSize → 327, dauerFormation → 15.8745, dauerRecovery → 14.5216,
 delay → 3.68301, hatchTime → 17.6784, l1Molt → 32.2, l1Stage → 14.5216,
 l2dDelay → 7.57647, l2dStage → 16.4157, l2Molt → 41.0392, l2Stage → 8.83922,
 l3Molt → 50.5098, l3Stage → 9.47059, l4Molt → 62.5059, l4Stage → 11.9961,
 layTime → 3.78824, minL2dDelay → 3.15686, minL2dStage → 11.9961,
 progenyRate → 5.3, progenyStart → 64.4, λmax → 0.0675087}
```

(Local) In[48]:=
```
ds1 = {dauerStage → dauerRecovery + dauerFormation,
   dauerDelay → dauerRecovery + dauerFormation - l3Stage} /. rWT
```

(Local) Out[48]=
```
{dauerStage → 30.3961, dauerDelay → 20.9255}
```

(Local) In[49]:=
```
rWT = Union[rWT, ds1]
```

(Local) Out[49]=
```
{broodSize → 327, dauerDelay → 20.9255, dauerFormation → 15.8745,
 dauerRecovery → 14.5216, dauerStage → 30.3961, delay → 3.68301, hatchTime → 17.6784,
 l1Molt → 32.2, l1Stage → 14.5216, l2dDelay → 7.57647, l2dStage → 16.4157,
 l2Molt → 41.0392, l2Stage → 8.83922, l3Molt → 50.5098, l3Stage → 9.47059,
 l4Molt → 62.5059, l4Stage → 11.9961, layTime → 3.78824, minL2dDelay → 3.15686,
 minL2dStage → 11.9961, progenyRate → 5.3, progenyStart → 64.4, λmax → 0.0675087}
```

■ Estimation of discount rate

Barnes and Hodgkin reported that the weak *tra-3* mutant produced 499 instead of 327 sperm, and that this delayed production of eggs 2.6h. This implies a sperm production rate:

(Local) In[50]:=
```
t3r1 = {tra3BroodSize → 499, tra3Delay → 2.6}
```

(Local) Out[50]=
```
{tra3BroodSize → 499, tra3Delay → 2.6}
```

(Local) In[51]:=
```
sr1 = {spermRate → (tra3BroodSize - broodSize) / tra3Delay /. rWT /. t3r1}
```

(Local) Out[51]=
```
{spermRate → 66.1538}
```

Cutter 2004 measured sperm rate at 23.6 sperm/h.

(Local) In[52]:=
```
sr2 = {cutterSpermRate → 23.6}
```

(Local) Out[52]=
```
{cutterSpermRate → 23.6}
```

(Local) In[53]:=
```
rWT = Union[rWT, sr1, sr2]
```

(Local) Out[53]=
```
{broodSize → 327, cutterSpermRate → 23.6, dauerDelay → 20.9255,
 dauerFormation → 15.8745, dauerRecovery → 14.5216, dauerStage → 30.3961,
 delay → 3.68301, hatchTime → 17.6784, l1Molt → 32.2, l1Stage → 14.5216,
 l2dDelay → 7.57647, l2dStage → 16.4157, l2Molt → 41.0392, l2Stage → 8.83922,
 l3Molt → 50.5098, l3Stage → 9.47059, l4Molt → 62.5059, l4Stage → 11.9961,
 layTime → 3.78824, minL2dDelay → 3.15686, minL2dStage → 11.9961,
 progenyRate → 5.3, progenyStart → 64.4, spermRate → 66.1538, λmax → 0.0675087}
```

Here's the progeny production rate as a function of age for the wild-type.

(Local) In[54]:=
```
progeny[a_] :=
  progenyRate UnitStep[a - progenyStart, progenyStart + broodSize / progenyRate - a]
```

Suppose sperm are produced for time st longer than the wild-type case. Then progeny production will start at time progenyStart + st, and it will continue for (broodSize + st × spermRate) / progenyRate.

(Local) In[55]:=
```
progeny[a_, st_] := progenyRate UnitStep[a - (progenyStart + st),
   progenyStart + st + (broodSize + st × spermRate) / progenyRate - a]
```

(Local) In[56]:=
```
Plot[Evaluate[{progeny[a, 0], progeny[a, 2.6]} /. rWT], {a, 0, 200}]
```

(Local) Out[56]=

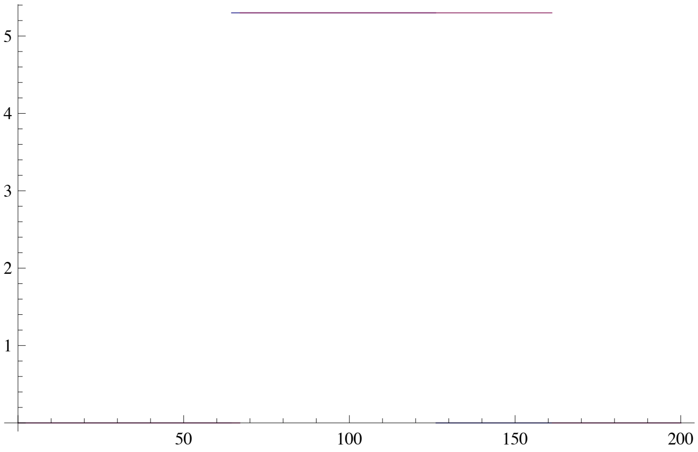

(Local) In[57]:=
```
npv[st_, λ_] := Evaluate[
    Simplify[-1 + ∫_0^∞ (progeny[a, st] /. rWT) ⅇ^(-λ a) ⅆa, broodSize + st spermRate > 0 /. rWT]]
```

(Local) In[58]:=
```
npv[st, λ]
```

(Local) Out[58]= $-1+\frac{5.3\left(-e^{(-126.098-13.4819\,\mathrm{st})\,\lambda}+e^{(-64.4-1.\,\mathrm{st})\,\lambda}\right)}{\lambda}$

(The assumption broodSize + st×spermRate > 0 is the condition that SOME sperm have to be produced.)

(Local) In[59]:=
```
Plot[Evaluate[Table[npv[st, λ], {λ, 0.03, 0.07, 0.01}]], {st, -5, 5}]
```

(Local) Out[59]=
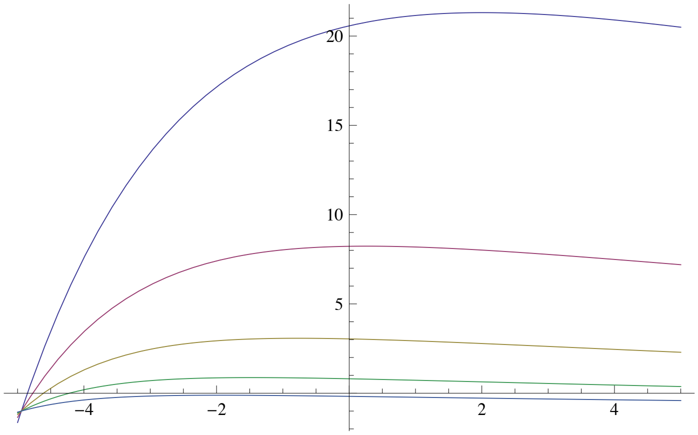

This is a plot of NPV vs st at various discount rates. As you would expect, the maximum occurs at larger values of st for smaller discount rates λ. The max is close to the wild-type value 0 for λ = 0.04, which suggests that is the discount rate for which wild-type is optimized. The exact sperm time that maximizes NPV at discount rate λ is calculated as follows:

(Local) In[60]:=
```
Solve[D[npv[st, λ], st] == 0, st][[1, 1]]
```

Solve::ifun : Inverse functions are being used by Solve, so
some solutions may not be found; use Reduce for complete solution information. ≫

(Local) Out[60]= $\mathrm{st}\to-\frac{0.0801163\,(-2.60134+61.6981\,\lambda)}{\lambda}$

(Local) In[61]:=
```
stmax[λ_] := Evaluate[st /. Solve[D[npv[st, λ], st] == 0, st][[1, 1]]]; stmax[λ]
```

Solve::ifun : Inverse functions are being used by Solve, so
some solutions may not be found; use Reduce for complete solution information. ≫

(Local) Out[61]= $-\frac{0.0801163\,(-2.60134+61.6981\,\lambda)}{\lambda}$

Now I can ask the key question: for what λ does the wild-type sperm time (0) maximize NPV?

(Local) In[62]:=
```
Solve[stmax[λhat] == 0, λhat][[1, 1]] // N
```

Solve::ratnz : Solve was unable to solve the system with inexact coefficients. The
answer was obtained by solving a corresponding exact system and numericizing the result. ≫

(Local) Out[62]= λhat → 0.0421625

This is a satisfying estimate. It is less than the lab growth rate 0.067, as it should be, but not enormously less, which seems reasonable. Neither of those things had to be true, so this is reassuring. From now on I'll use this as the discount rate when I need a numerical value.

(Local) In[63]:=
```
rWT = Union[rWT, Solve[stmax[λhat] == 0, λhat][[1]]]
```

Solve::ratnz : Solve was unable to solve the system with inexact coefficients. The answer was obtained by solving a corresponding exact system and numericizing the result. ≫

(Local) Out[63]=
```
{broodSize → 327, cutterSpermRate → 23.6, dauerDelay → 20.9255,
 dauerFormation → 15.8745, dauerRecovery → 14.5216, dauerStage → 30.3961,
 delay → 3.68301, hatchTime → 17.6784, l1Molt → 32.2, l1Stage → 14.5216,
 l2dDelay → 7.57647, l2dStage → 16.4157, l2Molt → 41.0392, l2Stage → 8.83922,
 l3Molt → 50.5098, l3Stage → 9.47059, l4Molt → 62.5059, l4Stage → 11.9961,
 layTime → 3.78824, minL2dDelay → 3.15686, minL2dStage → 11.9961, progenyRate → 5.3,
 progenyStart → 64.4, spermRate → 66.1538, λhat → 0.0421625, λmax → 0.0675087}
```

(Local) In[64]:=
```
Log[2] / λhat /. rWT
```

(Local) Out[64]= 16.4399

What happens if I use Asher Cutter's measured sperm rate?

(Local) In[65]:=
```
rcWT = DeleteCases[rWT, spermRate → _] /. cutterSpermRate → spermRate
```

(Local) Out[65]=
```
{broodSize → 327, spermRate → 23.6, dauerDelay → 20.9255,
 dauerFormation → 15.8745, dauerRecovery → 14.5216, dauerStage → 30.3961,
 delay → 3.68301, hatchTime → 17.6784, l1Molt → 32.2, l1Stage → 14.5216,
 l2dDelay → 7.57647, l2dStage → 16.4157, l2Molt → 41.0392, l2Stage → 8.83922,
 l3Molt → 50.5098, l3Stage → 9.47059, l4Molt → 62.5059, l4Stage → 11.9961,
 layTime → 3.78824, minL2dDelay → 3.15686, minL2dStage → 11.9961,
 progenyRate → 5.3, progenyStart → 64.4, λhat → 0.0421625, λmax → 0.0675087}
```

(Local) In[66]:=
```
npvc[st_, λ_] := Evaluate[
  Simplify[-1 + ∫_0^∞ (progeny[a, st] /. rcWT) e^(-λ a) da, broodSize + st spermRate > 0 /. rcWT]]
```

(Local) In[67]:=
```
{npv[st, λ], npvc[st, λ]}
```

(Local) Out[67]=

$$\left\{-1+\frac{5.3\left(-e^{(-126.098-13.4819\,st)\,\lambda}+e^{(-64.4-1.\,st)\,\lambda}\right)}{\lambda},\ -1+\frac{5.3\left(-e^{(-126.098-5.45283\,st)\,\lambda}+e^{(-64.4-1.\,st)\,\lambda}\right)}{\lambda}\right\}$$

(Local) In[68]:=
```
Plot[Evaluate[Table[npvc[st, λ], {λ, 0.03, 0.07, 0.01}]], {st, -10, 10}]
```

(Local) Out[68]=

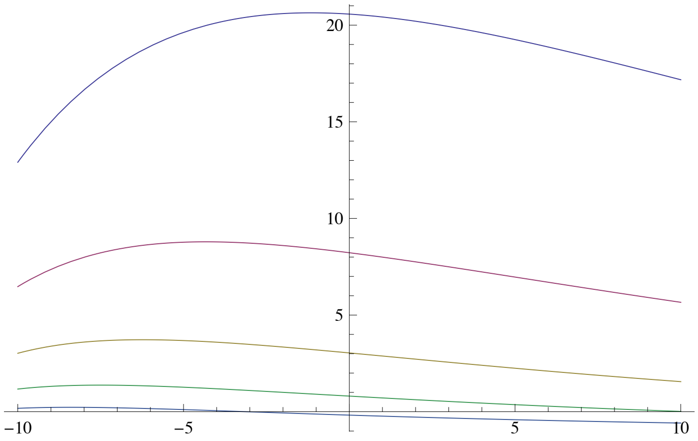

(Local) In[69]:=
```
Solve[D[npvc[st, λ], st] == 0, st][[1, 1]]
```

Solve::ifun : Inverse functions are being used by Solve, so some solutions may not be found; use Reduce for complete solution information. ≫

(Local) Out[69]=

$$st \to -\frac{0.224576\,(-1.69613+61.6981\,\lambda)}{\lambda}$$

(Local) In[70]:= **stmaxc[λ_] := Evaluate[st /. Solve[D[npvc[st, λ], st] == 0, st][[1, 1]]]; stmaxc[λ]**

Solve::ifun : Inverse functions are being used by Solve, so
some solutions may not be found; use Reduce for complete solution information. ≫

(Local) Out[70]= $-\frac{0.224576\ (-1.69613 + 61.6981\ \lambda)}{\lambda}$

(Local) In[71]:= **Solve[stmaxc[λcutter] == 0, λcutter][[1, 1]] // N**

Solve::ratnz : Solve was unable to solve the system with inexact coefficients. The
answer was obtained by solving a corresponding exact system and numericizing the result. ≫

(Local) Out[71]= λcutter → 0.0274909

(Local) In[72]:= **rWT = Union[rWT, Solve[stmaxc[λcutter] == 0, λcutter][[1]] // N]**

Solve::ratnz : Solve was unable to solve the system with inexact coefficients. The
answer was obtained by solving a corresponding exact system and numericizing the result. ≫

(Local) Out[72]= {broodSize → 327, cutterSpermRate → 23.6, dauerDelay → 20.9255, dauerFormation → 15.8745,
dauerRecovery → 14.5216, dauerStage → 30.3961, delay → 3.68301,
hatchTime → 17.6784, l1Molt → 32.2, l1Stage → 14.5216, l2dDelay → 7.57647,
l2dStage → 16.4157, l2Molt → 41.0392, l2Stage → 8.83922, l3Molt → 50.5098,
l3Stage → 9.47059, l4Molt → 62.5059, l4Stage → 11.9961, layTime → 3.78824,
minL2dDelay → 3.15686, minL2dStage → 11.9961, progenyRate → 5.3, progenyStart → 64.4,
spermRate → 66.1538, λcutter → 0.0274909, λhat → 0.0421625, λmax → 0.0675087}

(Local) In[73]:= **Log[2] / λcutter /. rWT**

(Local) Out[73]= 25.2137

▪ *tra-3* calculation

(Local) In[74]:= **pv1[λt3, progenyStart + $\frac{499 - \text{broodSize}}{\text{progenyRate}}$, progenyRate, 499] /. rWT**

(Local) Out[74]= $\frac{5.3\ e^{-191.004\ \lambda t3}\left(-1 + e^{94.1509\ \lambda t3}\right)}{\lambda t3}$

(Local) In[75]:= **FindRoot[1 == pv1[λt3, progenyStart + $\frac{499 - \text{broodSize}}{\text{spermRate}}$, progenyRate, 499] /. rWT, {λt3, 0.05}]**

(Local) Out[75]= {λt3 → 0.0655343}

(Local) In[76]:= **rWT = Union[rWT, FindRoot[
1 == pv1[λt3, progenyStart + $\frac{499 - \text{broodSize}}{\text{spermRate}}$, progenyRate, 499] /. rWT, {λt3, 0.05}]]**

(Local) Out[76]= {broodSize → 327, cutterSpermRate → 23.6, dauerDelay → 20.9255, dauerFormation → 15.8745,
dauerRecovery → 14.5216, dauerStage → 30.3961, delay → 3.68301, hatchTime → 17.6784,
l1Molt → 32.2, l1Stage → 14.5216, l2dDelay → 7.57647, l2dStage → 16.4157,
l2Molt → 41.0392, l2Stage → 8.83922, l3Molt → 50.5098, l3Stage → 9.47059,
l4Molt → 62.5059, l4Stage → 11.9961, layTime → 3.78824, minL2dDelay → 3.15686,
minL2dStage → 11.9961, progenyRate → 5.3, progenyStart → 64.4, spermRate → 66.1538,
λcutter → 0.0274909, λhat → 0.0421625, λmax → 0.0675087, λt3 → 0.0655343}

(Local) In[77]:= **λmax / λt3 /. rWT**

(Local) Out[77]= 1.03013

WT eggs produced before *tra-3* starts, WT and *tra-3* eggs produced together, *tra-3* eggs produced after WT ends, together with times in hours of these periods.

(Local) In[78]:=
```
Outer[Times, {(499 - broodSize)/spermRate,
   broodSize/progenyRate - (499 - broodSize)/spermRate,
   (499 - broodSize)/spermRate + 499/progenyRate - broodSize/progenyRate
  }, {1, progenyRate}] /. rWT
```

(Local) Out[78]=
```
{{2.6, 13.78}, {59.0981, 313.22}, {35.0528, 185.78}}
```

Values

The dauer is the unit of account. A dauer can recover and in time dauerRecovery become an L4, so the value of the L4 (right after the dauer or L3 molt) is:

(Local) In[79]:=
```
l4Value → ⅇ^(λhat dauerRecovery) /. rWT
```

(Local) Out[79]=
```
l4Value → 1.84461
```

(Local) In[80]:=
```
rWT = Union[rWT, {dauerValue → 1.0, l4Value → ⅇ^(λhat dauerRecovery) /. rWT}];
```

Other stages are based on that. Note that the l4Value is value at time l3Molt, which is why that appears in all the expression below. yaValue is the value at the L4 molt.

(Local) In[81]:=
```
{l1Value → l4Value ⅇ^(-l3Molt λhat),
  l2Value → l4Value ⅇ^((l1Molt-l3Molt) λhat),
  l3Value → l4Value ⅇ^((l2Molt-l3Molt) λhat),
  yaValue → l4Value ⅇ^((l4Molt-l3Molt) λhat)} /. rWT
```

(Local) Out[81]=
```
{l1Value → 0.219292, l2Value → 0.852381, l3Value → 1.23734, yaValue → 3.05888}
```

(Local) In[82]:=
```
rWT = Union[rWT, {l1Value → l4Value ⅇ^(-l3Molt λhat),
     l2Value → l4Value ⅇ^((l1Molt-l3Molt) λhat),
     l3Value → l4Value ⅇ^((l2Molt-l3Molt) λhat),
     yaValue → l4Value ⅇ^((l4Molt-l3Molt) λhat)} /. rWT];
```

Finally, there is a complication with the dauer. When an L2d molts to become a dauer, the value is not immediately 1. That's because it needs time dauerFormation to actually become a dauer. In competition with an L2d that has just decided to become an L3, it will need time dauerFormation + dauerRecovery to become an L4, so it is worth less than a dauer and much less than an L3.

(Local) In[83]:=
```
{newDauerValue → ⅇ^(-λhat dauerFormation) /. rWT}
```

(Local) Out[83]=
```
{newDauerValue → 0.512063}
```

(Local) In[84]:=
```
rWT = Union[rWT, {newDauerValue → ⅇ^(-λhat dauerFormation) /. rWT}]
```

(Local) Out[84]=
```
{broodSize → 327, cutterSpermRate → 23.6, dauerDelay → 20.9255, dauerFormation → 15.8745,
  dauerRecovery → 14.5216, dauerStage → 30.3961, dauerValue → 1., delay → 3.68301,
  hatchTime → 17.6784, l1Molt → 32.2, l1Stage → 14.5216, l1Value → 0.219292,
  l2dDelay → 7.57647, l2dStage → 16.4157, l2Molt → 41.0392, l2Stage → 8.83922,
  l2Value → 0.852381, l3Molt → 50.5098, l3Stage → 9.47059, l3Value → 1.23734,
  l4Molt → 62.5059, l4Stage → 11.9961, l4Value → 1.84461, layTime → 3.78824,
  minL2dDelay → 3.15686, minL2dStage → 11.9961, newDauerValue → 0.512063,
  progenyRate → 5.3, progenyStart → 64.4, spermRate → 66.1538, yaValue → 3.05888,
  λcutter → 0.0274909, λhat → 0.0421625, λmax → 0.0675087, λt3 → 0.0655343}
```

■ Growth and size

(Local) In[85]:=
```
<< PhysicalConstants`
```

General::obspkg :
PhysicalConstants` is now obsolete. The legacy version being loaded may conflict with current Mathematica functionality. See the Compatibility Guide for updating information.

Volumes of worms (from Knight CG, Patel MN, Azevedo RBR & Leroi AM (2002) A novel mode of ecdysozoan growth in Caenorhabditis elegans. Evolution & Development 4: 16-27.). They say "volume (V, in $\mu m^3 \times 10^3$) increases exponentially with time (t, in hours): $V = 46.6 \exp(0.0722\, t)$." Using data Armand Leroi sent me, I got $85.3 \exp(0.0716\, t)$ (leroi_v1.nb). They give the times of the molts as L1: 13.6h, L2: 22.2h, L3: 29.2h, L4: 39.9h (from Hirsh et al, 1976).

(Local) In[86]:=
```
ck25to20 = (l4Molt - hatchTime) / 39.9 /. rWT
```

(Local) Out[86]=
```
1.1235
```

(Local) In[87]:=
```
λgrowth → 0.07160528853285601` / ck25to20
```

(Local) Out[87]=
```
λgrowth → 0.0637344
```

Very close to λmax, which is encouraging.

(Local) In[88]:=
```
rWT = Union[rWT, {λgrowth → 0.07160528853285601` / ck25to20}]
```

(Local) Out[88]=
```
{broodSize → 327, cutterSpermRate → 23.6, dauerDelay → 20.9255,
 dauerFormation → 15.8745, dauerRecovery → 14.5216, dauerStage → 30.3961,
 dauerValue → 1., delay → 3.68301, hatchTime → 17.6784, l1Molt → 32.2,
 l1Stage → 14.5216, l1Value → 0.219292, l2dDelay → 7.57647, l2dStage → 16.4157,
 l2Molt → 41.0392, l2Stage → 8.83922, l2Value → 0.852381, l3Molt → 50.5098,
 l3Stage → 9.47059, l3Value → 1.23734, l4Molt → 62.5059, l4Stage → 11.9961,
 l4Value → 1.84461, layTime → 3.78824, minL2dDelay → 3.15686,
 minL2dStage → 11.9961, newDauerValue → 0.512063, progenyRate → 5.3,
 progenyStart → 64.4, spermRate → 66.1538, yaValue → 3.05888, λcutter → 0.0274909,
 λgrowth → 0.0637344, λhat → 0.0421625, λmax → 0.0675087, λt3 → 0.0655343}
```

(Local) In[89]:=
```
{kL1m, kL2m, kL3m, kL4m} = {13.6, 22.2, 29.2, 39.9} Hour
```

(Local) Out[89]=
```
{13.6 Hour, 22.2 Hour, 29.2 Hour, 39.9 Hour}
```

(Local) In[90]:=
```
kV1[t_] := 46 600. Exp[0.0722 t / Hour] (Micro Meter)^3
```

(Local) In[91]:=
```
Convert[kV1[#], Liter] & /@ {0, kL1m, kL2m, kL3m, kL4m, 44 Hour}
```

(Local) Out[91]=
$\{4.66 \times 10^{-11}$ Liter, 1.24402×10^{-10} Liter, 2.31468×10^{-10} Liter, 3.83692×10^{-10} Liter, 8.30793×10^{-10} Liter, 1.117×10^{-9} Liter$\}$

An egg is about $30 \times 30 \times 50 = 45\,000\ \mu m^3$, which is consistent with the $t = 0$ volume.

In Fig 5 the adults range from 1,500,000 to 6,300,000 μm^3 (1.5 - 6.3 nL). A nice round figure for a grA might be 3,000,000.

(Local) In[92]:=
```
vGrA = Convert[3 000 000. (Micro Meter)^3, Liter]
```

(Local) Out[92]=
$3. \times 10^{-9}$ Liter

(Local) In[93]:=
```
grAMass = N[Convert[vGrA (1 Kilogram / Liter), Gram]]
```

(Local) Out[93]=
$3. \times 10^{-6}$ Gram

Number of doublings to reach Earth mass:

(Local) In[94]:=
```
Log[2, SI[EarthMass / grAMass]]
```

(Local) Out[94]=
```
110.617
```

How many doublings would it take for the descendants of an adult to reach Earth mass at the lab growth rate?

(Local) In[95]:=
```
Convert[Log[SI[EarthMass / grAMass]] / λmax Hour /. rWT, #] & /@ {Day, Week, Month, Year}
```

(Local) Out[95]=
```
{47.3237 Day, 6.76052 Week, 1.55585 Month, 0.129654 Year}
```

(Local) In[96]:=
```
Log[SI[EarthMass / grAMass]] / Quantity[10^6, "Years"]
```

(Local) Out[96]=
```
0.0000766741 per year
```

(Local) In[97]:=
```
Log[SI[EarthMass / grAMass]] / Quantity[10^6, "Years"] * Quantity[6, "Months"]
```

(Local) Out[97]=
```
0.0000383371
```

(Local) In[98]:=
```
With[{r = Log[SI[EarthMass / grAMass]] / Quantity[10^6, "Years"] * Quantity[6, "Months"]},
  {e^-r, e^r}]
```

(Local) Out[98]=
```
{0.999962, 1.00004}
```

100x increase in 100 years:

(Local) In[99]:=
```
With[{r = Log[100.] / Quantity[100, "Years"] * Quantity[6, "Months"]},
  {r, e^-r, e^r}
 ]
```

(Local) Out[99]=
```
{0.0230259, 0.977237, 1.02329}
```

How many times its volume does an adult produce in eggs?

(Local) In[100]:=
```
vEgg = kV1[0]
```

(Local) Out[100]= 46 600. $\text{Meter}^3\ \text{Micro}^3$

(Local) In[101]:=
```
broodSize vEgg / vGrA /. rWT // SI
```

(Local) Out[101]=
```
5.0794
```

A dauer is probably about the same size as a new L3:

(Local) In[102]:=
```
vDauer = Convert[kV1[kL2m], Liter]
```

(Local) Out[102]= 2.31468×10^{-10} Liter

An apple, according to Wikipedia, is typically 5-9 cm diameter. Taking the smaller figure as more conservative and perhaps more typical of wild apples, that's a volume of roughly

(Local) In[103]:= vApple = Convert$\left[\frac{4}{3} \pi \left(\frac{5.\ \text{Centi Meter}}{2}\right)^3, \text{Liter}\right]$

(Local) Out[103]=
```
0.0654498 Liter
```

(Local) In[104]:= $\frac{\text{vDauer}}{\text{vApple}}$

(Local) Out[104]= 3.53657×10^{-9}

If a worm managed to produce 10^5 dauers, that would capture roughly 0.03% of the biomass (well, volume, really) of a small apple.

(Local) In[105]:= $10^5 \frac{\text{vDauer}}{\text{vApple}}$

(Local) Out[105]=
```
0.000353657
```

A crude estimate of gonad volume as a proportion of the adlt hermaphrodite: Let's model the gonad as a cylinder of

diameter 40% that of the worm. Doubled up, it runs, let's say, 75% the length. Modeling the worm also as a cylinder of diameter d and length l, the gonad occupies:

(Local) In[106]:=
$$\frac{2\times 0.75\, l \times (0.4\, d)^2}{l\, d^2}$$

(Local) Out[106]= 0.24

Let's call it 1/4.

Binomal model

The environment is either good with probability p or bad with probability $1 - p$. In a good environment, the L3 is worth V; in bad, 0. The dauer is worth 1 independent of environment. The L3 value is pV.

(Local) In[107]:=
```
biv1[p_, V_] := p V
```

(Local) In[108]:=
```
p (V - biv1[p, V])^2 + (1 - p) biv1[p, V]^2 // Simplify
```

(Local) Out[108]= $-(-1+p)\, p\, V^2$

(Local) In[109]:=
```
bivar1[p_, V_] := Evaluate[
   p (V - biv1[p, V])^2 + (1 - p) biv1[p, V]^2 // Simplify
  ];
bivar1[p, V]
```

(Local) Out[110]= $-(-1+p)\, p\, V^2$

(Local) In[111]:=
```
$Assumptions = 0 < p < 1 && σ > 0 && V > 0 && μ > 0
```

(Local) Out[111]= $0 < p < 1 \;\&\&\; \sigma > 0 \;\&\&\; V > 0 \;\&\&\; \mu > 0$

(Local) In[112]:=
```
bieq1 = {μ == biv1[p, V], σ^2 == bivar1[p, V]}
```

(Local) Out[112]= $\{\mu == p\, V,\ \sigma^2 == -(-1+p)\, p\, V^2\}$

(Local) In[113]:=
```
Solve[bieq1, {p, V}][[1]] // Simplify
```

(Local) Out[113]= $\left\{p \to \frac{\mu^2}{\mu^2+\sigma^2},\ V \to \mu + \frac{\sigma^2}{\mu}\right\}$

(Local) In[114]:=
```
bivar1[1/#, #] & /@ {1.25, 2, 10}
```

(Local) Out[114]= {0.25, 1, 9}

To hedge the option, I need a dauers and b L3s satisfying:

(Local) In[115]:=
```
bieq2 = {a + 0 b == 1, a + b V == V}
```

(Local) Out[115]= {a == 1, a + b V == V}

(Local) In[116]:=
```
Solve[bieq2, {a, b}][[1]]
```

(Local) Out[116]= $\left\{a \to 1,\ b \to 1 - \frac{1}{V}\right\}$

The value of the L2d is

(Local) In[117]:=
$$1 + \left(1 - \frac{1}{V}\right) p\, V \text{ // Simplify}$$

(Local) Out[117]= $1 + p\,(-1 + V)$

If L3 value $pV = 1$, then L2d value is:

(Local) In[118]:= $1 + \left(1 - \frac{1}{V}\right) p V \;/.\; p \to \frac{1}{V}$ // Simplify

(Local) Out[118]= $2 - \frac{1}{V}$

and this can be assigned to the worm (1) and the option $\left(1 - \frac{1}{V}\right)$. This is sensible because the dauer and the L3 have the same value. If they were different, it wouldn't be obvious how to break the value down into the worm and the option.

Calculations for specific examples:

(Local) In[119]:=
```
rWT
```

(Local) Out[119]=
```
{broodSize → 327, cutterSpermRate → 23.6, dauerDelay → 20.9255,
 dauerFormation → 15.8745, dauerRecovery → 14.5216, dauerStage → 30.3961,
 dauerValue → 1., delay → 3.68301, hatchTime → 17.6784, l1Molt → 32.2,
 l1Stage → 14.5216, l1Value → 0.219292, l2dDelay → 7.57647, l2dStage → 16.4157,
 l2Molt → 41.0392, l2Stage → 8.83922, l2Value → 0.852381, l3Molt → 50.5098,
 l3Stage → 9.47059, l3Value → 1.23734, l4Molt → 62.5059, l4Stage → 11.9961,
 l4Value → 1.84461, layTime → 3.78824, minL2dDelay → 3.15686,
 minL2dStage → 11.9961, newDauerValue → 0.512063, progenyRate → 5.3,
 progenyStart → 64.4, spermRate → 66.1538, yaValue → 3.05888, λcutter → 0.0274909,
 λgrowth → 0.0637344, λhat → 0.0421625, λmax → 0.0675087, λt3 → 0.0655343}
```

(Local) In[120]:=
```
m`l2
```

(Local) Out[120]=
```
m`l2
```

(Local) In[121]:=
```
binomTable1[p_, v_, l2Stage_: m`l2Stage, l2dStage_: m`l2dStage, λ_: m`λ] :=
 Module[{heads, dauerRow, l2Row, l2dRow, l2Discount, l2dDiscount},
  l2Discount = e^(-l2Stage λ);
  l2dDiscount = e^(-l2dStage λ);
  heads = {"State", "Bad", "Good", "Mean", "Bad", "Good", "Mean"};
  dauerRow = {"dauer", 1, 1, 1, l2dDiscount, l2dDiscount, l2dDiscount};
  l2Row = {"L2", 0, v, p v, 0, v l2Discount, p v l2Discount};
  l2dRow =
   {"L2d", 1, v, p v + (1 - p), l2dDiscount, v l2dDiscount, l2dDiscount (p v + 1 - p)};
  {heads, dauerRow, l2Row, l2dRow}
 ]
```

(Local) In[122]:=
```
Grid[binomTable1[0.667, 1.5, 9, 16]]
```

(Local) Out[122]=

State	Bad	Good	Mean	Bad	Good	Mean
dauer	1	1	1	0.50936	0.50936	0.50936
L2	0	1.5	1.0005	0	1.02634	0.684571
L2d	1	1.5	1.3335	0.50936	0.764041	0.679232

(Local) In[123]:=
```
Grid[binomTable1[0.5, 2, 9, 16]]
```

(Local) Out[123]=

State	Bad	Good	Mean	Bad	Good	Mean
dauer	1	1	1	0.50936	0.50936	0.50936
L2	0	2	1.	0	1.36846	0.684229
L2d	1	2	1.5	0.50936	1.01872	0.764041

(Local) In[124]:=
```
Grid[binomTable1[0.8, 1.25, 9, 16]]
```

(Local) Out[124]=

State	Bad	Good	Mean	Bad	Good	Mean
dauer	1	1	1	0.50936	0.50936	0.50936
L2	0	1.25	1.	0	0.855287	0.684229
L2d	1	1.25	1.2	0.50936	0.6367	0.611232

(Local) In[125]:=
```
Grid[binomTable1[0.1, 10, 9, 16]]
```

(Local) Out[125]=

State	Bad	Good	Mean	Bad	Good	Mean
dauer	1	1	1	0.50936	0.50936	0.50936
L2	0	10	1.	0	6.84229	0.684229
L2d	1	10	1.9	0.50936	5.0936	0.967785

Variance and upside

Are there any bounds that can be placed on the probability of high value based on variance alone? Value must be positive. I will assume here that the mean is 1, since that can be scaled to get results for any other mean.

Let $\mathrm{Var}(v) = \sigma^2$. Divide possible results into those above b and those below. Let $p = \mathbb{P}[v > b]$, $q = 1 - p$. Now, I think I can partition the variance. Let $f(v)\, dv$ be the PDF of v. Define μ_- and σ_-^2 as the mean and variance of v's below b, μ_+ and σ_+^2 as the mean and variance of v's above b:

$$\sigma_-^2 = \frac{1}{q} \int_0^b f(v)\,(v - \mu_-)^2\, dv$$

$$\int_0^b f(v)\,(v - \mu_-)^2\, dv = \int_0^b f(v)\, v^2\, dv - \int_0^b 2\, f(v)\, v \mu_-\, dv + \int_0^b f(v)\, \mu_-^2\, dv$$

$$= \int_0^b f(v)\, v^2\, dv - 2\, \mu_- \int_0^b f(v)\, v dv + \mu_-^2 \int_0^b f(v)\, dv$$

$$= \int_0^b f(v)\, v^2\, dv - 2\, q\mu_-^2 + q\mu_-^2$$

$$= \int_0^b f(v)\, v^2\, dv - q\mu_-^2$$

$$\sigma_-^2 = \frac{1}{q} \int_0^b f(v)\, v^2\, dv - \mu_-^2$$

Likewise:

$$\sigma_+^2 = \frac{1}{p} \int_b^\infty f(v)\, v^2\, dv - \mu_+^2.$$

Then:

$$\sigma^2 = \int_0^\infty f(v)\, v^2\, dv - 1$$

$$= \int_0^b f(v)\, v^2\, dv + \int_b^\infty f(v)\, v^2\, dv - 1$$

$$= q\left(\sigma_-^2 + \mu_-^2\right) + p\left(\sigma_+^2 + \mu_+^2\right) - 1$$

$$= q\sigma_-^2 + q\mu_-^2 + p\sigma_+^2 + p\mu_+^2 - 1$$

$$p(\mu_+ - 1)^2 + q(\mu_- - 1)^2 = p\mu_+^2 - 2\, p\mu_+ + p + q\mu_-^2 - 2\, q\mu_- + q$$

$$= p\mu_+^2 + q\mu_-^2 - 2\,(p\mu_+ + q\mu_-) + p + q$$

Now, $p + q = 1$, and $(p\mu_+ + q\mu_-) = \mu = 1$. So,

$$p(\mu_+ - 1)^2 + q(\mu_- - 1)^2 = p\mu_+^2 + q\mu_-^2 - 2 \times 1 + 1$$

$$= p\mu_+^2 + q\mu_-^2 - 1$$

So, finally,

$$\sigma^2 = p\sigma_+^2 + q\sigma_-^2 + p(\mu_+ - 1)^2 + q(\mu_- - 1)^2.$$

$\sigma_-^2 \le \frac{b^2}{4}$. But there's no limit to how big σ_+^2 can get, even while keeping μ_+ as close to b as I like. So I guess there's no limit on how small p can get. Rats.

But I do have another constraint: $(p\mu_+ + q\mu_-) = 1$. Let's suppose that v is distributed so as to maximize σ_-^2. That'll be b with probability $\frac{\mu_-}{b}$, 0 with probability $1 - \frac{\mu_-}{b}$:

$$\sigma_-^2 = \left(1-\frac{\mu_-}{b}\right)\left(\frac{\mu_-}{b}\right)^2 + \frac{\mu_-}{b}\left(1-\frac{\mu_-}{b}\right)^2$$
$$= \left(1-\frac{\mu_-}{b}\right)\left(\frac{\mu_-}{b}\right)\left(\left(\frac{\mu_-}{b}\right) + \left(1-\frac{\mu_-}{b}\right)\right)$$
$$= \left(1-\frac{\mu_-}{b}\right)\left(\frac{\mu_-}{b}\right).$$

Nope, still no dice. I can have $p = \delta$, $\mu_+ = b + \epsilon$, for δ and ϵ as small as I like, and still make things work out by making μ_- as close to 1 as necessary and σ_+^2 as big as necessary. There's an *upper* bound—I can't have $pb > 1$—but no lower bound. The only thing I can set a lower bound on is σ_+^2, but that's obvious, and has no direct selective significance.

Black-Scholes model

■ Formulas

```
(Local) In[126]:= wormOptionsDir = "H:\\science\\worm_options"

(Local) Out[126]= H:\science\worm_options

(Local) In[127]:= AppendTo[$Path, wormOptionsDir];

(Local) In[128]:= << itoCalculus_v6.m

(Local) In[129]:= << Shreve.m

(Local) In[130]:= itoSetup[{t}, {W1, W2}, 0 ≤ t ≤ T]

(Local) Out[130]= 0 ≤ t ≤ T

(Local) In[131]:= $Assumptions = Union[$Assumptions && α > 0 && ν > 0 && σ > 0]

(Local) Out[131]= α > 0 && ν > 0 && σ > 0 && 0 ≤ t ≤ T
```

$s(t)$ is the "stock price", the value of a standard reproductive larva. $v(t)$ is the value of an option on s. It depends on t and $s(t)$.

```
(Local) In[132]:= setItoD[{v, s}]

(Local) Out[132]= {Null, Null}
```

s is a geometric Brownian motion with volatility σ.

```
(Local) In[133]:= rds = ⅆ[s[t], t] → σ s[t] ⅆ[W2, t]

(Local) Out[133]= ⅆ s[t] → σ s[t] ⅆ W2[t]

(Local) In[134]:= itoD[v[t, s[t]]] /. rds /. s[t] → s // itoSimplify // itoCollect
```

(Local) Out[134]= $s\,\sigma\, v^{(0,1)}[t,s]\, ⅆ W2[t] + \left(\frac{1}{2} s^2 \sigma^2 v^{(0,2)}[t,s] + v^{(1,0)}[t,s]\right) ⅆ t$

v is a martingale, so the $ⅆ t$ coefficient must vanish.

```
(Local) In[135]:= bspdf1 =
    Coefficient[itoD[v[t, s[t]]] /. rds /. s[t] → s // itoSimplify, itoDifferential[t]]
```

(Local) Out[135]= $\frac{1}{2} s^2 \sigma^2 v^{(0,2)}[t,s] + v^{(1,0)}[t,s]$

Boundary conditions. These are not directly useful as written since they're too tricky for *Mathematica*.

(Local) In[136]:=
```
bsbc1 = {
   v[-∞, s] == 0,
   v[t, 0] == 0,
   Limit[v[t, s], s → ∞] == s,
   v[T, s] == Max[1, s]
  }
```

(Local) Out[136]= {v[-∞, s] == 0, v[t, 0] == 0, Limit[v[t, s], s → ∞] == s, v[T, s] == Max[1, s]}

(Local) In[137]:= bsypdf1 = bspdf1 /. $\left\{v \to \text{Function}\left[\{t, s\}, y\left[T - t, \text{Log}[s] - \frac{\sigma^2 (T - t)}{2}\right]\right]\right\}$ /. $\left\{T - t \to \tau, \text{Log}[s] - \frac{\sigma^2 (T - t)}{2} \to u\right\}$ // Simplify

(Local) Out[137]= $\frac{1}{2}\sigma^2 y^{(0,2)}[\tau, u] - y^{(1,0)}[\tau, u]$

That's the heat equation, and it has a Gaussian solution:

(Local) In[138]:= PDF[NormalDistribution[y0, $\sigma\sqrt{\tau}$], u]

(Local) Out[138]= $\frac{e^{-\frac{(u-y0)^2}{2\sigma^2\tau}}}{\sqrt{2\pi}\,\sigma\sqrt{\tau}}$

(Local) In[139]:= bsypdf1 /. y → Function[{τ, u}, Evaluate[PDF[NormalDistribution[y0, $\sigma\sqrt{\tau}$], u]]] // Simplify

(Local) Out[139]= 0

(Local) In[140]:=
```
ys1[τ_, u_, u0_] := Evaluate[
    PDF[NormalDistribution[u0, σ √τ], u]
  ];
ys1[τ, u, u0]
```

(Local) Out[141]= $\frac{e^{-\frac{(u-u0)^2}{2\sigma^2\tau}}}{\sqrt{2\pi}\,\sigma\sqrt{\tau}}$

Now, I should be able to convolve that with max(1, s) to get the solution. At $\tau = 0$

$v(T - \tau, s) = y(0, \log s) = \max(1, s)$
$y(0, u) = \max(1, e^u)$.

(Local) In[142]:= $Assumptions = Union[$Assumptions && τ > 0 && σ > 0]

(Local) Out[142]= α > 0 && ν > 0 && σ > 0 && τ > 0 && 0 ≤ t ≤ T

A couple checks:

(Local) In[143]:= $\left\{\int_{-\infty}^{\infty} \text{ys1}[\tau, u, u0]\, du0, \int_{-\infty}^{\infty} \text{ys1}[\tau, u, u0]\, e^{u0}\, du0\right\}$

(Local) Out[143]= $\left\{1, e^{u+\frac{\sigma^2\tau}{2}}\right\}$

(Local) In[144]:= $\int_{-\infty}^{\infty} \text{ys1}[\tau, u, u0]\, \text{Max}\left[1, e^{u0}\right] du0$ // noErf

(Local) Out[144]= $\frac{1}{2}\left(e^{u+\frac{\sigma^2\tau}{2}} + 2\,\text{normalCDF}\left[-\frac{u}{\sigma\sqrt{\tau}}\right] + e^{u+\frac{\sigma^2\tau}{2}}\left(-1 + 2\,\text{normalCDF}\left[\frac{u+\sigma^2\tau}{\sigma\sqrt{\tau}}\right]\right)\right)$

(Local) In[145]:= **bssol1 =** $\int_{-\infty}^{\infty}$ **ys1[τ, u, u0] Max[1, ⅇ^u0] ⅆu0 /. u → Log[s] -** $\frac{\sigma^2 \tau}{2}$ **// noErf // Simplify**

(Local) Out[145]= $\text{normalCDF}\left[\frac{\sigma^2 \tau - 2\,\text{Log}[s]}{2\,\sigma\sqrt{\tau}}\right] + s\,\text{normalCDF}\left[\frac{\frac{\sigma^2 \tau}{2} + \text{Log}[s]}{\sigma\sqrt{\tau}}\right]$

(Local) In[146]:= **bssol2 = bssol1 /. τ → T - t**

(Local) Out[146]= $\text{normalCDF}\left[\frac{(-t+T)\,\sigma^2 - 2\,\text{Log}[s]}{2\sqrt{-t+T}\,\sigma}\right] + s\,\text{normalCDF}\left[\frac{\frac{1}{2}(-t+T)\,\sigma^2 + \text{Log}[s]}{\sqrt{-t+T}\,\sigma}\right]$

(Local) In[147]:= **bssol2 /. normalCDF → Φ // TraditionalForm**

(Local) Out[147]//TraditionalForm=

$$\Phi\left(\frac{\sigma^2 (T-t) - 2\log(s)}{2\sigma\sqrt{T-t}}\right) + s\,\Phi\left(\frac{\log(s) + \frac{1}{2}\sigma^2 (T-t)}{\sigma\sqrt{T-t}}\right)$$

That looks right. For large *s* the first term is 0, the second is 1, and you get *s*. For small s, the first term is 1, the second is 0, and you get 1.

(Local) In[148]:= **bspdf1 /. v → Function[{t, s}, Evaluate[bssol2]]**

(Local) Out[148]=

$$\frac{e^{-\frac{((-t+T)\sigma^2 - 2\,\text{Log}[s])^2}{8(-t+T)\sigma^2}}\left(-\frac{\sigma}{2\sqrt{-t+T}} + \frac{(-t+T)\sigma^2 - 2\,\text{Log}[s]}{4(-t+T)^{3/2}\sigma}\right)}{\sqrt{2\pi}} +$$

$$\frac{1}{2}s^2\sigma^2\left(\frac{e^{-\frac{((-t+T)\sigma^2-2\,\text{Log}[s])^2}{8(-t+T)\sigma^2}}}{\sqrt{2\pi}\,s^2\sqrt{-t+T}\,\sigma} + \frac{e^{-\frac{(\frac{1}{2}(-t+T)\sigma^2+\text{Log}[s])^2}{2(-t+T)\sigma^2}}}{\sqrt{2\pi}\,s\sqrt{-t+T}\,\sigma} - \frac{e^{-\frac{((-t+T)\sigma^2-2\,\text{Log}[s])^2}{8(-t+T)\sigma^2}}\left((-t+T)\sigma^2 - 2\,\text{Log}[s]\right)}{2\sqrt{2\pi}\,s^2(-t+T)^{3/2}\sigma^3} - \frac{e^{-\frac{(\frac{1}{2}(-t+T)\sigma^2+\text{Log}[s])^2}{2(-t+T)\sigma^2}}\left(\frac{1}{2}(-t+T)\sigma^2 + \text{Log}[s]\right)}{\sqrt{2\pi}\,s\,(-t+T)^{3/2}\sigma^3}\right) + \frac{e^{-\frac{(\frac{1}{2}(-t+T)\sigma^2+\text{Log}[s])^2}{2(-t+T)\sigma^2}}\,s\left(-\frac{\sigma}{2\sqrt{-t+T}} + \frac{\frac{1}{2}(-t+T)\sigma^2+\text{Log}[s]}{2(-t+T)^{3/2}\sigma}\right)}{\sqrt{2\pi}}$$

(Local) In[149]:= **bspdf1 /. v → Function[{t, s}, Evaluate[bssol2]] // Simplify**

(Local) Out[149]= 0

Check.

(Local) In[150]:= **bsf1[τ_, s_, σ_] :=**
Evaluate[bssol1]

(Local) In[151]:= **rWT**

(Local) Out[151]= {broodSize → 327, cutterSpermRate → 23.6, dauerDelay → 20.9255,
dauerFormation → 15.8745, dauerRecovery → 14.5216, dauerStage → 30.3961,
dauerValue → 1., delay → 3.68301, hatchTime → 17.6784, l1Molt → 32.2,
l1Stage → 14.5216, l1Value → 0.219292, l2dDelay → 7.57647, l2dStage → 16.4157,
l2Molt → 41.0392, l2Stage → 8.83922, l2Value → 0.852381, l3Molt → 50.5098,
l3Stage → 9.47059, l3Value → 1.23734, l4Molt → 62.5059, l4Stage → 11.9961,
l4Value → 1.84461, layTime → 3.78824, minL2dDelay → 3.15686,
minL2dStage → 11.9961, newDauerValue → 0.512063, progenyRate → 5.3,
progenyStart → 64.4, spermRate → 66.1538, yaValue → 3.05888, λcutter → 0.0274909,
λgrowth → 0.0637344, λhat → 0.0421625, λmax → 0.0675087, λt3 → 0.0655343}

(Local) In[152]:=
```
bsf1[l2dStage /. rWT, 1, 1]
```

(Local) Out[152]= 2 normalCDF[2.02581]

- **Gadget**

(Local) In[153]:=
```
Module[{minσsT = -1.5, maxσsT = 1.0},
 Manipulate[
  Module[{σ, τ, σsT = 10^lσsT, is = 400},
   Grid[{
     {Show[Graphics[Style[Text["σ√T  = " <> ToString[NumberForm[σsT, 2]]],
          Bold, FontSize → 12, FontFamily -> "Helvetica"]],
       ImageSize → {Full, 15}]},
     {Plot[{bsf1[σsT^2, s, 1], s, 1}, {s, 0, 2},
       PlotStyle → {Directive[Red, Thick],
         Directive[Darker[Green, 0.7], Dashed], Directive[Black, Dashed]},
       Epilog → {
         Style[Text["L2d", {1, N[bsf1[σsT^2, 1, 1]]}, {1, -1}],
          Bold, Red, FontSize → 12, FontFamily → "Helvetica"],
         Style[Text["dauer", {1.5, 1}, {0, -1}], Bold, Black,
          FontSize → 12, FontFamily → "Helvetica"],
         Style[Text["L3", {0.5, 0.5}, {0, -1}], Bold, Darker[Green, 0.7],
          FontSize → 12, FontFamily → "Helvetica"]
        },
       PlotRange → {{0, 2}, {0, 3}},
       Frame → {{True, False}, {True, False}},
       FrameStyle → Directive[Bold, Black, FontSize → 12, FontFamily → "Helvetica"],
       FrameLabel → {"predicted environment quality", "value"},
       ImageSize → is, AspectRatio → 1]}
     }]
   ],
  {{lσsT, minσsT, "σ√T "}, minσsT, maxσsT}
  ]
 ]
```

(Local) Out[153]=

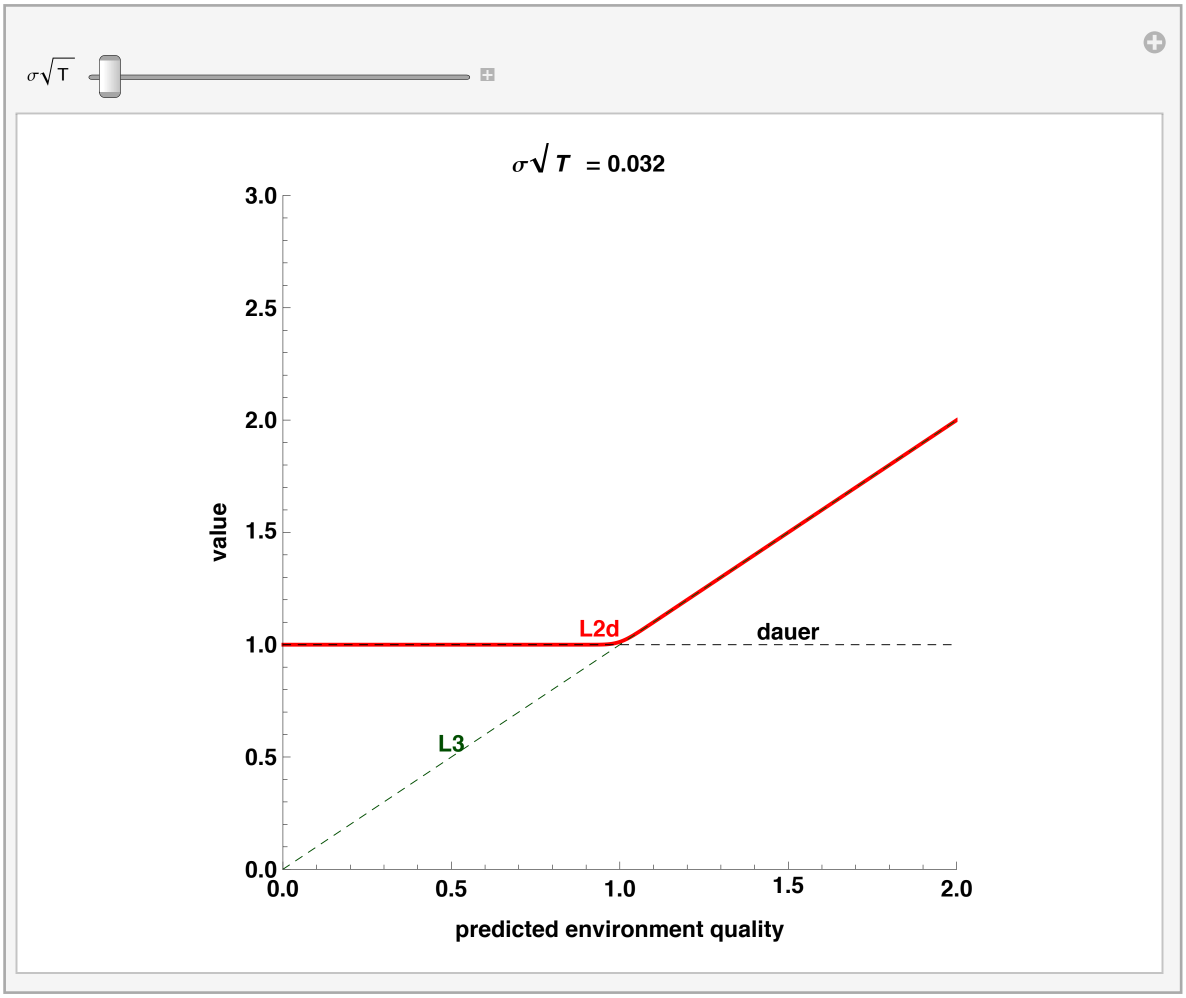

▪ Plot

(Local) In[154]:=
```
Module[{σ, τ, σ2τs = {0.2, 3}, is = 400, pfs, ps, lfs = 10},
 pfs = {s, 1, Max[1, s], 1 + s}~Join~
   (bsf1[#, s, 1] & /@ σ2τs);
 ps = {Directive[Darker[Green, 0.5], AbsoluteThickness[5], Dashed],
    Directive[Gray, AbsoluteThickness[5], Dashed],
    Directive[Black, AbsoluteThickness[2]],
    Directive[Black, AbsoluteThickness[2]]}~Join~
   ConstantArray[Directive[Black, AbsoluteThickness[1]], Length[σ2τs]];
 Plot[
  Evaluate[pfs], {s, 0, 2},
  PlotStyle → ps,
  Epilog →
   {Style[Text["dauer", {1.5, 1}, {0, 1}],
      Bold, Gray, FontSize → 12, FontFamily → "Helvetica"],
     Style[Text["L3", {0.5, 0.5}, {0, -1}], Bold, Darker[Green, 0.5],
      FontSize → 12, FontFamily → "Helvetica"],
     Style[Text["u = 0", {1.3, 1.15}, {-1, -1}], Bold, Black,
      FontSize → lfs, FontFamily → "Helvetica"],
     Style[Text["u = ∞", {1.5, 2.5}, {1, -1}], Bold, Black,
      FontSize → lfs, FontFamily → "Helvetica"]}
    ~Join~
    (Style[Text["u = " <> ToString[#], {1.5, N[bsf1[#, 1.5, 1]]}, {1, -1}],
        Bold, Black, FontSize → lfs, FontFamily → "Helvetica"] & /@ σ2τs),
  Frame → {{True, False}, {True, False}},
  FrameStyle → Directive[Bold, Black, FontSize → 12, FontFamily → "Helvetica"],
  FrameLabel → {"predicted environment quality", "value"},
  PlotRange → {{0, 2}, {0, 3}},
  ImageSize → is, AspectRatio → 1]
]
```

(Local) Out[154]=

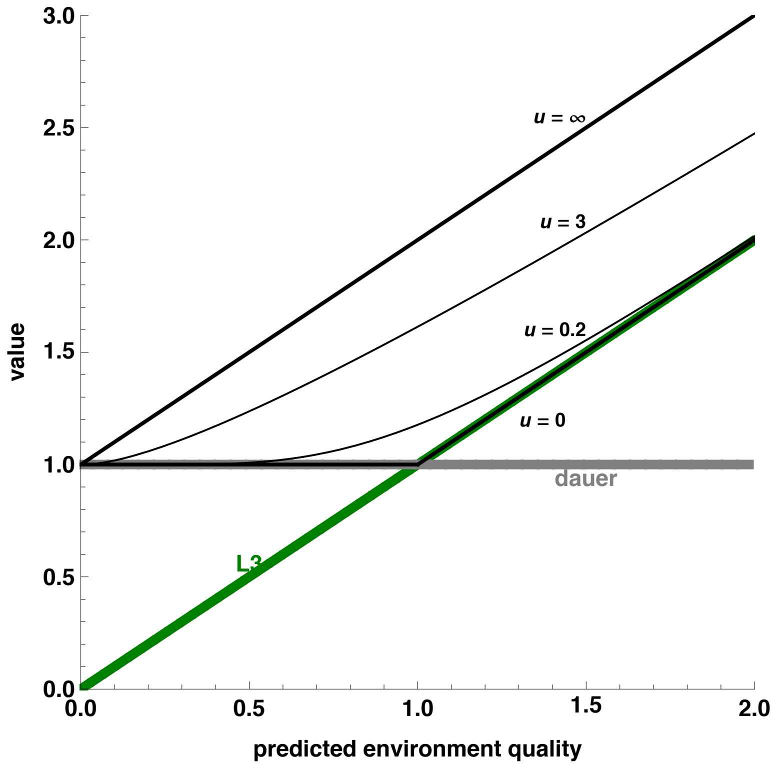

Supplement: Dataset S1 — Calculations. Calculations were done in Wolfram Mathematica. This dataset contains Mathematica notebooks that carry out the calculations and explain them in detail. There are two principle notebooks, amer_model_v6.nb and calculations_v2.nb. Files init_v3.m, init_v3.nb, itoCalculus_v6.m, itoCalculus_v6.nb, and Shreve.m contain supporting code necessary for amer_model_v6.nb and calculations_v2.nb to evaluate. PDF printouts of the two main notebooks are also provided so that they can be read without Mathematica. (ZIP) [file pone.0100580.s001.zip › calcs/calculations_v2.pdf]
