# Supplementary material for: A Model of the Effect of Uncertainty on the C elegans L2/L2d Decision
Source: PLoS One. 2014 Jul 16;9(7):e100580. doi: 10.1371/journal.pone.0100580 (PMC4100763; doi:10.1371/journal.pone.0100580)
Supplement: Dataset S1 — Calculations. Calculations were done in Wolfram Mathematica. This dataset contains Mathematica notebooks that carry out the calculations and explain them in detail. There are two principle notebooks, amer_model_v6.nb and calculations_v2.nb. Files init_v3.m, init_v3.nb, itoCalculus_v6.m, itoCalculus_v6.nb, and Shreve.m contain supporting code necessary for amer_model_v6.nb and calculations_v2.nb to evaluate. PDF printouts of the two main notebooks are also provided so that they can be read without Mathematica. (ZIP) [file pone.0100580.s001.zip › calcs/amer_model_v6.pdf]

Setup

(Local) In[1]:=
```
{NotebookFileName[], DateString[]}
```

(Local) Out[1]=
```
{/Volumes/leon Home/papers/return/calcs/amer_model_v6.nb, Mon 3 Mar 2014 07:37:01}
```

(Local) In[2]:=
```
Get[FileNameJoin[{FileNameDrop[NotebookFileName[]], "init_v3.m"}]]
```

(Local) In[3]:=
```
rWT
```

(Local) Out[3]=
```
{broodSize → 327, cutterSpermRate → 23.6, dauerDelay → 20.9255, dauerFormation → 15.8745,
 dauerRecovery → 14.5216, dauerStage → 30.3961, dauerValue → 1., delay → 3.68301,
 hatchTime → 17.6784, l1Molt → 32.2, l1Stage → 14.5216, l1Value → 0.219292,
 l2dDelay → 7.57647, l2dStage → 16.4157, l2Molt → 41.0392, l2Stage → 8.83922,
 l2Value → 0.852381, l3Molt → 50.5098, l3Stage → 9.47059, l3Value → 1.23734,
 l4Molt → 62.5059, l4Stage → 11.9961, l4Value → 1.84461, layTime → 3.78824,
 minL2dDelay → 3.15686, minL2dStage → 11.9961, newDauerValue → 0.512063, progenyRate → 5.3,
 progenyStart → 64.4, spermRate → 66.1538, yaValue → 3.05888, λcutter → 0.0274909,
 λgrowth → 0.0637344, λhat → 0.0421625, λmax → 0.0675087, λt3 → 0.0655343}
```

(Local) In[4]:=
```
params
```

(Local) Out[4]=
```
{α → 0.0569294, a → 8.83922, λ → 0.0421625, δ → 0.538462, dValue → 0.512063}
```

(Local) In[5]:=
```
Begin["amer`"]
```

(Local) Out[5]=
```
amer`
```

Functions

■ Summary of European value computation

To summarize what I've got so far, the value of the European L2d is

$$v(a, q) = e^{\frac{\lambda a}{\delta}} (V_d + V_{L3}\, q) - e^{\frac{\lambda a}{2\delta}} \sqrt{V_d V_{L3}\, q}\; y\left(a, \log\left(\frac{V_{L3}}{V_q} q\right)\right)$$

$$v(a, q) = V_d\, e^{\frac{\lambda a}{2\delta}} \left(e^{\frac{\lambda a}{2\delta}} (1 + e^u) - e^{u/2}\, y(a, u)\right)$$

$$u = \log\left(\frac{V_{L3}}{V_q} q\right), q = \frac{V_d}{V_{L3}} e^u$$

$$v(a, q) = e^{\frac{\lambda}{2\delta} a} \sqrt{V_d V_{L3}\, q}\; y_1(a, u)$$

$$y_1(a, u) = y_{\max}(a, u) - y(a, u)$$

$$y_{\max}(a, u) = e^{\frac{\lambda}{2\delta} a} \left(e^{-u/2} + e^{u/2}\right)$$

The L2d molt is defined as $a = 0$, so $a < 0$ in all the computations below.

$$y(0, u) = y_0(u) = e^{-|u|/2}$$

$y(a, u)$ is calculated by convolving $y_0(u)$ with a kernel whose Fourier transform is $e^{\kappa(\omega)\, a}$, with $\kappa(\omega) = \sqrt{\frac{\lambda(2\,\alpha\lambda + \sigma^2 + 4\,\sigma^2\,\omega^2)}{8\,\alpha\delta^2}}$.

(Local) In[6]:=
```
κ[ω_, α_, λ_, σ_, δ_] :=
```
$$\sqrt{\frac{\lambda\,(2\,\alpha\,\lambda + \sigma^2 + 4\,\sigma^2\,\omega^2)}{8\,\alpha\,\delta^2}}$$

L2 value, L2d value, and intrinsic value

The value of the L2 is $V_{L3}\, q e^{\lambda a} = V_d\, e^{u+\lambda a}$.

(Local) In[7]:=
```
l2v1[a_, u_, α_, λ_, σ_, δ_, dValue_, l3Value_] :=
  dValue e^(u+λ a);
l2v1[a, u, α, λ, σ, δ, dValue, l3Value]
```

(Local) Out[8]= $\text{dValue}\, e^{u+a\lambda}$

(Local) In[9]:=
```
l2v2[a_, q_, α_, λ_, σ_, δ_, dValue_, l3Value_] :=
  Evaluate[
   l2v1[a, Log[l3Value/dValue q], α, λ, σ, δ, dValue, l3Value]
  ];
l2v2[a, q, α, λ, σ, δ, dValue, l3Value]
```

(Local) Out[10]= $e^{a\lambda}\, \text{l3Value}\, q$

The value of a (hypothetical) L2d commited to become a dauer is $V_d\, e^{\frac{\lambda}{\delta} a}$.

(Local) In[11]:=
```
l2dv1[a_, u_, α_, λ_, σ_, δ_, dValue_, l3Value_] :=
  dValue e^(λ a/δ);
l2dv1[a, u, α, λ, σ, δ, dValue, l3Value]
```

(Local) Out[12]= $\text{dValue}\, e^{\frac{a\lambda}{\delta}}$

(Local) In[13]:=
```
l2dv2[a_, q_, α_, λ_, σ_, δ_, dValue_, l3Value_] :=
  dValue e^(λ a/δ);
l2dv2[a, q, α, λ, σ, δ, dValue, l3Value]
```

(Local) Out[14]= $\text{dValue}\, e^{\frac{a\lambda}{\delta}}$

The intrinsic value of the L2d is the maximum of these two.

(Local) In[15]:=
```
l2div1[a_, u_, α_, λ_, σ_, δ_, dValue_, l3Value_] :=
  Max[l2dv1[a, u, α, λ, σ, δ, dValue, l3Value], l2v1[a, u, α, λ, σ, δ, dValue, l3Value]];
l2div1[a, u, α, λ, σ, δ, dValue, l3Value]
```

(Local) Out[16]= $\text{Max}\left[\text{dValue}\, e^{\frac{a\lambda}{\delta}}, \text{dValue}\, e^{u+a\lambda}\right]$

(Local) In[17]:=
```
l2div2[a_, q_, α_, λ_, σ_, δ_, dValue_, l3Value_] :=
  Evaluate[
   l2div1[a, Log[l3Value/dValue q], α, λ, σ, δ, dValue, l3Value]
  ];
l2div2[a, q, α, λ, σ, δ, dValue, l3Value]
```

(Local) Out[18]= $\text{Max}\left[\text{dValue}\, e^{\frac{a\lambda}{\delta}}, e^{a\lambda}\, \text{l3Value}\, q\right]$

American value computation

■

For this I want to use a different $y_{\max}$ that will track with intrinsic value. This will keep $y(a, u)$ within bounds during the solution. However, since it will no longer be a solution of the PDE, I'm going to end up with an inhomogeneous correction. Actually, to avoid confusion, let's use $w_{\max}$ and w for these new transformations. I start with the same transformation as before:

$v(a, q) = e^{\frac{\lambda}{2\delta} a} \sqrt{V_d V_{L3} q}\; w_1(a, u)$

w_1 satisfies the transformed PDE

$0 = -\lambda\left(2\,\alpha\lambda + \sigma^2\right) w_1 + 4\,\lambda\sigma^2\, w_{1uu} + 8\,\alpha\,\delta^2\, w_{1aa}$

Now, let

$v_{\max}(a, u) = V_d\, e^{\frac{\lambda}{\delta} a} + V_{L3}\, q e^{\lambda a} = V_d\left(e^{\frac{\lambda}{\delta} a} + e^{u+\lambda a}\right)$,

$v_{\max}(a, q) = e^{\frac{\lambda}{2\delta} a} \sqrt{V_d V_{L3} q}\; w_{\max}(a, u)$,

(same transformation as for $v \to w_1$),

$w_{\max}(a, u) = e^{-\frac{\lambda}{2\delta} a}\left(e^{\frac{\lambda}{\delta} a}\, e^{-u/2} + e^{\lambda a}\, e^{u/2}\right)$

$w(a, u) = w_{\max}(a, u) - w_1(a, u)$.

Let's suppose that at some particular value of $a = a_0$ I have a $w(a_0, u)$ that is a solution. I want to take a step to $a_0 - h$.

Define $\hat{w}(a_0, u) = w(a_0, u)$. Convolve $\hat{w}(a_0, u)$ with a kernel whose FT is $e^{-\kappa(\omega)\, h}$, with $\kappa(\omega) = \sqrt{\frac{\lambda\left(2\,\alpha\lambda+\sigma^2+4\,\sigma^2\,\omega^2\right)}{8\,\alpha\delta^2}}$. This gives me a $\hat{w}(a_0 - h, u)$ such that $\hat{w}$ solves the same PDE as w_1. Let

$\hat{w}_{\max}(a_0 - h, u) = e^{\frac{\lambda}{2\delta}(a_0 - h)}\, e^{-u/2} + e^{-\frac{\lambda}{2\delta} h}\, e^{\left(1-\frac{1}{2\delta}\right)\lambda a_0}\, e^{u/2}$.

- **Verification**

(Local) In[19]:= $\texttt{vpde2} = \frac{1}{2} q^2\, \sigma^2\, \partial_{q,q} v[a, q] - \alpha\,\delta\, \partial_a v[a, q] + \frac{\alpha\,\delta^2\, \partial_{a,a} v[a, q]}{\lambda}$

(Local) Out[19]= $\frac{1}{2} q^2\, \sigma^2\, v^{(0,2)}[a, q] - \alpha\,\delta\, v^{(1,0)}[a, q] + \frac{\alpha\,\delta^2\, v^{(2,0)}[a, q]}{\lambda}$

(Local) In[20]:= $\texttt{ypde1} = \frac{\texttt{vpde2}}{e^{\frac{\lambda}{2\delta} a} \sqrt{q}}$ /. v → Function$\left[\{a, q\}, e^{\frac{\lambda}{2\delta} a} \sqrt{q}\; y\left[a, \mathrm{Log}\left[\frac{\texttt{l3Value}}{\texttt{dValue}} q\right]\right]\right]$ /. $\mathrm{Log}\left[\frac{\texttt{l3Value}}{\texttt{dValue}} q\right] \to u$ // cFunc[y]

(Local) Out[20]= $\frac{1}{8}\left(-2\,\alpha\,\lambda - \sigma^2\right) y[a, u] + \frac{1}{2} \sigma^2\, y^{(0,2)}[a, u] + \frac{\alpha\,\delta^2\, y^{(2,0)}[a, u]}{\lambda}$

(Local) In[21]:= $\texttt{wmax[a\_, u\_]} := e^{-\frac{\lambda}{2\delta} a}\left(e^{\frac{\lambda}{\delta} a}\, e^{-u/2} + e^{\lambda a}\, e^{u/2}\right)$

(Local) In[22]:= ypde1 /. y → Function[{a, u}, wmax[a, u]] // Simplify

(Local) Out[22]= $e^{\frac{1}{2}\left(u + \frac{a(-1+2\delta)\lambda}{\delta}\right)}\, \alpha\, (-1 + \delta)\, \delta\, \lambda$

(Local) In[23]:= $\texttt{whmax[h\_, u\_]} := e^{\frac{\lambda}{2\delta}(a0 - h)}\, e^{-u/2} + e^{-\frac{\lambda}{2\delta} h}\, e^{\left(1-\frac{1}{2\delta}\right)\lambda\, a0}\, e^{u/2}$

(Local) In[24]:= whmax[0, u] == wmax[a0, u] // Simplify

(Local) Out[24]= True

(Local) In[25]:= ypde1 /. y → Function[{a, u}, whmax[a0 - a, u]] // Simplify

(Local) Out[25]= 0

```
(Local) In[26]:= wmax[a0 - h, u] - whmax[h, u] // Simplify
```

(Local) Out[26]= $e^{\frac{1}{2}\left(u-\frac{(a0+h-2\,a0\,\delta)\,\lambda}{\delta}\right)}\left(-1+e^{h\left(-1+\frac{1}{\delta}\right)\lambda}\right)$

(Local) In[27]:= $\left(e^{\lambda a_0-\frac{\lambda}{2\delta}a_0-\frac{\lambda}{2\delta}h}\left(e^{-\lambda h+\frac{\lambda}{\delta}h}-1\right)e^{u/2}\ /.\ a_0\to a0\right)$ `== wmax[a0 - h, u] - whmax[h, u] // Simplify`

(Local) Out[27]= `True`

■

This also solves the PDE. Since $\hat{w}(a_0-h,u)$ is a solution and $\hat{w}_{\max}(a_0-h,u)$ is a solution and the PDE is homogeneous, $\hat{w}_{\max}(a_0-h,u)-\hat{w}(a_0-h,u)$ is a solution, and since at $h=0$ this reduces to $w_{\max}(a_0,u)-w(a_0,u)=w_1(a_0,u)$, then it is *the* solution: $w_1(a_0-h,u)=\hat{w}_{\max}(a_0-h,u)-\hat{w}(a_0-h,u)$. Now, I need to compute $w(a_0-h,u)$.

$$
\begin{aligned}
w(a_0-h,u) &= w_{\max}(a_0-h,u)-w_1(a_0-h,u)\\
&= w_{\max}(a_0-h,u)-\left(\hat{w}_{\max}(a_0-h,u)-\hat{w}(a_0-h,u)\right)\\
&= \hat{w}(a_0-h,u)+w_{\max}(a_0-h,u)-\hat{w}_{\max}(a_0-h,u)\\
&= \hat{w}(a_0-h,u)+e^{-\frac{\lambda}{2\delta}(a_0-h)}\left(e^{\frac{\lambda}{\delta}(a_0-h)}e^{-u/2}+e^{\lambda(a_0-h)}e^{u/2}\right)-\left(e^{\frac{\lambda}{2\delta}(a_0-h)}e^{-u/2}+e^{-\frac{\lambda}{2\delta}h}e^{\left(1-\frac{1}{2\delta}\right)\lambda a_0}e^{u/2}\right)\\
&= \hat{w}(a_0-h,u)+e^{\lambda a_0-\frac{\lambda}{2\delta}a_0-\frac{\lambda}{2\delta}h}\left(e^{\left(\frac{\lambda}{\delta}-\lambda\right)h}-1\right)e^{u/2}
\end{aligned}
$$

This is straightforward to compute, although it looks messy. The correction always makes w bigger. $w_{\max}>y_{\max}$, and w is what you have to subtract before computing value, so it makes sense that w should grow. I'll have to be careful about precision in calculating $e^{\frac{\lambda}{2\delta}h}-1$ and $e^{\lambda\left(\frac{3}{2\delta}-1\right)h}-1$, but since those change only when the step size changes, I can use as much time as required to compute them accurately.

Next I need to set truncate w to intrinsic value (or actually, just L3 value, since it will always beat committed value without truncation). This will take the min of w and some $\psi(a,u)>0$ that I need to work out, so if the correction gets too big, it will get knocked down here. w should always be positive (because if it's negative that means $v>v_{\max}$), so the result of all this will be bounded. The value if the worm switches to L3 is

$$
\begin{aligned}
&v_{L3}(a,q)=V_{L3}\,q\,e^{\lambda a}\\
&v_{L3}(a,q)=e^{\frac{\lambda}{2\delta}a}\sqrt{V_d\,V_{L3}\,q}\;\;w_{1,L3}(a,u)\\
&w_{1,L3}(a,u)=e^{\left(\lambda-\frac{\lambda}{2\delta}\right)a}\,e^{u/2}\\
&w_{L3}(a,u)=w_{\max}(a,u)-w_{1,L3}(a,u)\\
&=e^{-\frac{\lambda}{2\delta}a}\left(e^{\frac{\lambda}{\delta}a}\,e^{-u/2}+e^{\lambda a}\,e^{u/2}\right)-e^{u/2}e^{\left(\lambda-\frac{\lambda}{2\delta}\right)a}\\
&=e^{\frac{\lambda}{2\delta}a}\,e^{-u/2}+e^{\left(\lambda-\frac{\lambda}{2\delta}\right)a}\,e^{u/2}-e^{\left(\lambda-\frac{\lambda}{2\delta}\right)a}\,e^{u/2}\\
&=e^{\frac{\lambda}{2\delta}a}\,e^{-u/2}.
\end{aligned}
$$

That works out rather simple, because $v_{\max}$ is the sum of committed dauer value and committed L3 value, so the difference between max and committed L3 is just going to be dauer.

Finally, I need to get $v(a,q)$ back from $w(a,u)$.

$$
v(a,q)=\left(e^{\frac{\lambda}{\delta}a}\,V_d+e^{\lambda a}\,V_{L3}\,q\right)-e^{\frac{\lambda}{2\delta}a}\sqrt{V_d\,V_{L3}\,q}\;\;w\!\left(a,\log\!\left(\frac{V_{L3}}{V_q}\,q\right)\right)
$$

Functions

▪ Convolution and steps

expm1[x, p] is $e^x - 1$, computed to precision p even if $|x| << 1$. p defaults to MachinePrecision, and if $p =$ MachinePrecision, expm1 returns a machine number.

(Local) In[28]:=
```
SetAttributes[expm1, Listable]
```

(Local) In[29]:=
```
expm1[x_?NumberQ, p_: MachinePrecision] :=
 N[
  Exp[SetPrecision[x, N[p]]] - 1,
  p
 ]
```

(Local) In[30]:=
```
With[{l = {10^-3., 10^-5., 10^-10., 10^-15.}},
 {expm1[l],
  Precision /@ expm1[l],
  MachineNumberQ /@ expm1[l]}
]
```

(Local) Out[30]=
```
{{0.0010005, 0.0000100001, 1. × 10^-10, 1. × 10^-15},
 {MachinePrecision, MachinePrecision, MachinePrecision, MachinePrecision},
 {True, True, True, True}}
```

(Local) In[31]:=
```
$Assumptions = Union[$Assumptions && u ∈ Reals && dValue > 0 && l3Value > 0]
```

(Local) Out[31]=
```
u ∈ Reals && dValue > 0 && l3Value > 0 && α > 0 && λ > 0 && ν > 0 && σ > 0 && 0 < δ < 1 && 0 ≤ t ≤ T
```

(Local) In[32]:=
```
wmax1[a_, u_] :=
```
$$e^{-\frac{\lambda}{2\delta} a}\left(e^{\frac{\lambda}{\delta} a} e^{-u/2} + e^{\lambda a} e^{u/2}\right)$$

Simple function to set up $e^{u/2}$ vector.

(Local) In[33]:=
```
eu2vec[umin_, ϵ_, n_] :=
  e^(#/2.) & /@ Range[umin, umin + (n - 1) ϵ, ϵ]
```

Return the Fourier transform of the kernel for a step of $\Delta a = -h$. (h should be positive.) The kernel extends from $-n\epsilon$ to $(n-1)\epsilon$.

(Local) In[34]:=
```
kert4[n_, h_, ϵ_, α_, λ_, σ_, δ_] :=
 Module[{umin = -(n ϵ)/2, ωmax = N[π/ϵ], dω = N[π/(n ϵ)], kpt, kp},
  kpt = N[Array[Exp[-h ϰ[(# - 1) dω - ωmax, α, λ, σ, δ]] &, 2 n]];
  kpt = RotateRight[kpt, n];
  kp = Re[InverseFourier[kpt]];
  kp[[n/2 + 1 ;; n]] = 0;
  Fourier[kp]
 ]
```

wc4vec[a, h, α, λ, δ, eu2v] computes the w correction

$$e^{\lambda a_0 - \frac{\lambda}{2\delta} a_0 - \frac{\lambda}{2\delta} h}\left(e^{\left(\frac{\lambda}{\delta} - \lambda\right) h} - 1\right) e^{u/2}$$

eu2v is the vector of values of $e^{u/2}$; wc4vec returns a vector of corresponding corrections.

(Local) In[35]:=
$$e^{\lambda\left(a - \frac{a+h}{2\delta}\right)}\left(\text{Exp}\left[\lambda\left(\frac{1}{\delta} - 1\right) h\right] - 1\right) e^{u/2} == e^{\lambda a_0 - \frac{\lambda}{2\delta} a_0 - \frac{\lambda}{2\delta} h}\left(e^{\left(\frac{\lambda}{\delta} - \lambda\right) h} - 1\right) e^{u/2} \text{ /. } a_0 \to a \text{ // Simplify}$$

(Local) Out[35]=
```
True
```

(Local) In[36]:=
```
wc4vec[a_, h_, α_, λ_, δ_, eu2v_] :=
  Module[{ef = expm1[λ (1/δ - 1) h]},
   e^(λ (a - (a+h)/(2 δ))) ef eu2v
  ]
```

ψ4vec[a, λ, δ, eu2v] computes the (w) intrinsic value

$$e^{\frac{\lambda}{2\delta} a}\, e^{-u/2}$$

eu2v is the vector of values of $e^{u/2}$; ψ4vec returns a vector of corresponding values of w.

(Local) In[37]:=
```
ψ4vec[a_, λ_, δ_, eu2v_] :=
  e^(λ/(2 δ) a) / eu2v
```

Utility function to take a lot of minima efficiently.

(Local) In[38]:=
```
SetAttributes[min4, Listable]
```

(Local) In[39]:=
```
min4[a_, b_] := Min[a, b]
```

(Local) In[40]:=
```
min4[Range[8], Range[8, 1, -1]]
```

(Local) Out[40]=
```
{1, 2, 3, 4, 4, 3, 2, 1}
```

American step. ya is the starting vector, extending from u_{min} to $u_{min} + (n - 1)\,\epsilon$. The step is from a to $a - h$; a should be negative, h positive. n and ϵ are as for kert4; α, λ, σ, δ are as usual. kert and eu2v, if provided, are the FT of the transform kernel and the $e^{u/2}$ vector, respectively. If these are given, u_{min}, h, and ϵ are not used, since those just serve in the construction of these vectors. If not provided, they are computed. If you're going to take many steps on the same grid, it is most efficient to calculate the vectors once, then provide them as arguments.

(Local) In[41]:=
```
astep4[wa_, umin_?NumberQ, h_?NumberQ, ϵ_?NumberQ, a_?NumberQ,
   α_?NumberQ, λ_?NumberQ, σ_?NumberQ, δ_?NumberQ, kert_: Null, eu2v_: Null] :=
  Module[{n = Length[wa], kt, ev, ah = a - h, wap, wahh, wah, ψvh},
   If[kert === Null, kt = kert4[n, h, ϵ, α, λ, σ, δ], kt := kert];
   If[eu2v === Null, ev = eu2vec[umin, ϵ, n], ev := eu2v];
   wap = Join[wa, -Reverse[wa]];
   wahh = Re[InverseFourier[kt Fourier[wap]]];
   wah = wahh[[1 ;; n]] + wc4vec[a, h, α, λ, δ, ev];
   ψvh = ψ4vec[ah, λ, δ, ev];
   min4[wah, ψvh]
  ]
```

European step. The arguments are almost identical to those for astep4, except that there is no eu2v. estep4 works on $y(a, u)$, not $w(a, u)$ (see above). This means that the projected results are not corrected for the change in the committed L3 value.

(Local) In[42]:=
```
estep4[ye_, umin_?NumberQ, h_?NumberQ, ϵ_?NumberQ, a_?NumberQ,
   α_?NumberQ, λ_?NumberQ, σ_?NumberQ, δ_?NumberQ, kert_: Null] :=
  Module[{n = Length[ye], kt, ev, yep, yeh, u, ah = a - h},
   If[kert === Null, kt = kert4[n, h, ϵ, α, λ, σ, δ], kt = kert];
   yep = Join[ye, -Reverse[ye]];
   yeh = Re[InverseFourier[kt Fourier[yep]]];
   yeh[[1 ;; n]]
  ]
```

■ Terminal value

Set up the terminal value vector $w_0(u) = e^{-|u|/2}$. The grid is chosen to cover at least from $q = \frac{1}{q_{max}}$ to q_{max}. r is the

maximum grid spacing. Return is the list $\{\vec{w}_0, u_{min}, \epsilon\}$. The grid is Range[$u_{min}$, $u_{min} + (n-1)\epsilon$, ϵ], with $n = \text{Length}[\vec{w}_0]$.

(Local) In[43]:=
```
w0[u_] := e^(-Abs[u]/2)
```

(Local) In[44]:=
```
w0v[qmax_?NumberQ, r_?NumberQ, dValue_: m`dValue, l3Value_: m`l3Value] :=
  Module[{fmax, umax, ε, n, w0v},
   fmax = N[qmax Max[l3Value/dValue, dValue/l3Value]];
   umax = 2 N[Log[fmax] + r];
   n = Ceiling[Log[2, umax / r]];
   n = 2^n;
   ε = N[umax / n];
   w0v = w0[(#1 - 1) ε - umax] & /@ Range[2 n];
   {w0v, -umax, ε}
  ]
```

American pricing

$f = \text{av7f}[\alpha, \lambda, \sigma, \delta, \text{dValue}, \text{l3Value}, \text{amin}, \text{qmax}, r]$ makes $f[a, q]$ the value of the L2d with American pricing. f is valid for $\frac{1}{\text{qmax}} \le q \le \text{qmax}$, $\text{amin} \le a \le 0$. r is the resolution in $\log q$. amin, qmax, and r default to $-\text{m`l2Stage} = -8.83922$, $10.^3$, and 0.01. Step size in the a dimension is determined adaptively, controlled by the following options:

(Local) In[45]:=
```
Options[wv7v] = Options[wv7f] = Options[av7f] = {
    StartingStepSize → 1 / 128.,
    PrecisionGoal → 3,
    AccuracyGoal → 3,
    sampleSpacing → 16,
    minSamples → 8
   }
```

(Local) Out[45]=
```
{StartingStepSize → 0.0078125, PrecisionGoal → 3,
 AccuracyGoal → 3, sampleSpacing → 16, minSamples → 8}
```

sampleGrid[n, ss, ms] creates a sampling grid for spot-checking a vector. n is the length of the vector to be sampled, ss is the sample spacing, and ms in the minimum number of samples—i.e., if sampling at spacing ss would give less than ms samples, the sample spacing is reduced.

(Local) In[46]:=
```
sampleGrid[n_Integer, ss_Integer, ms_Integer] :=
  Module[{ns, s, s0},
   ns = Min[Max[Floor[n / ss], ms], n];
   s = Floor[n / ns];
   s0 = 1 + Floor[(n - (ns - 1) s) / 2];
   Range[s0, s0 + (ns - 1) s, s]
  ]
```

Relative difference between two numbers.

(Local) In[47]:=
```
relativeError[a_Real, b_Real] :=
  If[a == b, 0, Abs[a - b] / Max[Abs /@ {a, b}]]
```

Given two vectors v_1 and v_2 of the same length and sampling grid sgrid, return the maximum absolute and relative errors at sample points.

(Local) In[48]:=
```
sampleError[sgrid_, v1_, v2_] :=
  Module[{rerr, aerr},
   aerr = Max[Abs[v1[[#]] - v2[[#]]] & /@ sgrid];
   rerr = Max[relativeError[v1[[#]], v2[[#]]] & /@ sgrid];
   {aerr, rerr}
  ]
```

wv7v is the workhorse for solving the American value problem. It constructs a terminal value vector at $a = 0$ (the L2d molt), then takes small steps backward in a. It returns the list {wv, agrid, ugrid, umin, ϵ, n, h}. wv is a matrix of computed w's: wv⟦i, j⟧ = w(agrid⟦i⟧, ugrid⟦j⟧). n is the number of elements in the u grid, also Length[wv⟦i⟧]. h is the last step size used—this can be used for continuing the solution to before a_{min} if necessary. The solution will go back at least to a_{min} (normally a little further, depending on how the last step aligns).

(Local) In[49]:=
```
wv7v[α_?NumberQ, λ_?NumberQ, σ_?NumberQ, δ_?NumberQ, dValue_?NumberQ,
   l3Value_?NumberQ, amin_: Null, qmax_: Null, r_: Null, opts : OptionsPattern[]] :=
  Module[{hmin, ag, pg, ss, ms, me, aminv, qmaxv, rv, wv, wvh1, wvh2,
     wv2h, umin, ϵ, n, h, ev, kt, kt2, ugrid, agrid, sgrid, a, aerr, rerr},
   hmin = OptionValue[StartingStepSize];
   ag = 10^-OptionValue[AccuracyGoal];
   pg = 10^-OptionValue[PrecisionGoal];
   ss = OptionValue[sampleSpacing];
   ms = OptionValue[minSamples];
   aminv = If[amin === Null, N[-m`l2Stage], amin];
   qmaxv = If[qmax === Null, 1000., qmax];
   rv = If[r === Null, 0.01, r];
   h = hmin;
   agrid = {0};
   wv = {{}};
   {wv⟦1⟧, umin, ϵ} = w0v[qmaxv, rv, dValue, l3Value];
   n = Length[wv⟦1⟧];
   ugrid = umin - ϵ + ϵ Range[n];
   sgrid = sampleGrid[n, ss, ms];
   ev = eu2vec[umin, ϵ, n];
   kt = kert4[n, h, ϵ, α, λ, σ, δ];
   kt2 = kert4[n, 2 h, ϵ, α, λ, σ, δ];
   For[a = 0, a ≥ aminv, Null,
    wvh1 = astep4[wv⟦-1⟧, umin, h, ϵ, a, α, λ, σ, δ, kt, ev];
    wvh2 = astep4[wvh1, umin, h, ϵ, a - h, α, λ, σ, δ, kt, ev];
    wv2h = astep4[wv⟦-1⟧, umin, 2 h, ϵ, a, α, λ, σ, δ, kt2, ev];
    a -= h; AppendTo[wv, wvh1]; AppendTo[agrid, a];
    a -= h; AppendTo[wv, wvh2]; AppendTo[agrid, a];
    {aerr, rerr} = sampleError[sgrid, wvh2, wv2h];
    If[aerr ≤ ag && rerr ≤ pg,
     h *= 2;
     kt = kt2;
     kt2 = kert4[n, 2 h, ϵ, α, λ, σ, δ];
    ]
   ];
   {wv, agrid, ugrid, umin, ϵ, n, h}
  ]
```

wv7f returns an InterpolatingFunction for $w(a, u)$ solving the American value problem. Arguments are the same as for wv7v, which it calls to actually solve the problem. It returns the list {wf, agrid, ugrid, umin, ϵ, n, h}. wf is an Interpolating Function wf[a, u] = $w(a, u)$. Other returns are as for wv7f.

(Local) In[50]:=
```
wv7f[α_?NumberQ, λ_?NumberQ, σ_?NumberQ, δ_?NumberQ, dValue_?NumberQ,
   l3Value_?NumberQ, amin_: Null, qmax_: Null, r_: Null, opts : OptionsPattern[]] :=
  Module[{wv, ugrid, agrid, sgrid, waf, umin, ϵ, n, h},
   {wv, agrid, ugrid, umin, ϵ, n, h} =
    wv7v[α, λ, σ, δ, dValue, l3Value, amin, qmax, r, opts];
   waf = ListInterpolation[wv, {agrid, ugrid}];
   {waf, agrid, ugrid, umin, ϵ, n, h}
  ]
```

av7f returns an InterpolatingFunction solving the American value problem. Arguments are the same as for wv7f, which it calls to actually solve the problem. It returns an Interpolating Function av[a, q] = $v(a, q)$.

(Local) In[51]:=
```
av7f[α_?NumberQ, λ_?NumberQ, σ_?NumberQ, δ_?NumberQ, dValue_?NumberQ,
  l3Value_?NumberQ, amin_: Null, qmax_: Null, r_: Null, opts : OptionsPattern[]] :=
 Module[{ugrid, agrid, waf, umaxi},
  {waf, agrid, ugrid} = wv7f[α, λ, σ, δ, dValue, l3Value, amin, qmax, r, opts][[1 ;; 3]];
  umaxi = Min[-ugrid[[1]], ugrid[[-1]]];
  With[{wfv = waf, umax = umaxi, qmin = ⅇ^-umaxi, ϰ0 = ϰ[0, α, λ, 0, δ]},
   Function[{a, q}, av7if[a, q, dValue, l3Value, wfv, umax, qmin, ϰ0, λ]]
  ]
 ]
```

(Local) In[52]:=
```
av7if[a_, q_, dValue_, l3Value_, waf_, umax_, qmin_, ϰ0_, λ_] :=
 Module[{u = If[q > 0, Log[(q l3Value)/dValue], -∞], au,
   qn = dValue/l3Value ⅇ^-umax, qx = dValue/l3Value ⅇ^umax, dfd = ⅇ^(ϰ0 a), dfL3 = ⅇ^(λ a), vf, qv, uv},
  au = Abs[u];
  vf = Function[{qv, uv},
    dfd^2 dValue + dfL3 l3Value qv - dfd Sqrt[dValue l3Value qv] waf[a, uv]
   ];
  Which[u < -umax,
   Interpolation[{{qn, vf[qn, -umax]}, {0, dfd^2 dValue}}, qn, InterpolationOrder → 1],
   u > umax, dfL3 q,
   True, vf[q, u]
  ]
 ]
```

■ Delay of early exercise

(Local) In[53]:=
```
Solve[delay/m`δ + (m`l2Stage - delay) == m`minL2dStage, delay][[1]]
```

(Local) Out[53]= {delay → 3.68301}

(Local) In[54]:=
```
rWT = Union[rWT, Solve[delay/m`δ + (m`l2Stage - delay) == m`minL2dStage, delay][[1]]]
```

(Local) Out[54]=
```
{broodSize → 327, cutterSpermRate → 23.6, dauerDelay → 20.9255,
 dauerFormation → 15.8745, dauerRecovery → 14.5216, dauerStage → 30.3961,
 dauerValue → 1., delay → 3.68301, hatchTime → 17.6784, l1Molt → 32.2,
 l1Stage → 14.5216, l1Value → 0.219292, l2dDelay → 7.57647, l2dStage → 16.4157,
 l2Molt → 41.0392, l2Stage → 8.83922, l2Value → 0.852381, l3Molt → 50.5098,
 l3Stage → 9.47059, l3Value → 1.23734, l4Molt → 62.5059, l4Stage → 11.9961,
 l4Value → 1.84461, layTime → 3.78824, minL2dDelay → 3.15686,
 minL2dStage → 11.9961, newDauerValue → 0.512063, progenyRate → 5.3,
 progenyStart → 64.4, spermRate → 66.1538, yaValue → 3.05888, λcutter → 0.0274909,
 λgrowth → 0.0637344, λhat → 0.0421625, λmax → 0.0675087, λt3 → 0.0655343}
```

(Local) In[55]:=
```
m`delay = delay /. Solve[delay/m`δ + (m`l2Stage - delay) == m`minL2dStage, delay][[1]]
```

(Local) Out[55]= 3.68301

(Local) In[56]:=
```
{m`delay/m`δ, m`delay, m`delay/m`δ - m`delay, m`minL2dDelay}
```

(Local) Out[56]= {6.83987, 3.68301, 3.15686, 3.15686}

Hybrid Euro/American pricing

European + American option value computation. The worm is assumed to have decided at the L1 molt to become an L2d. It must remain an L2d until its age has increased by *delay*. After that it may switch to the L2 track at any time, and if it does so, it develops at rate α instead of $\delta\alpha$. (This is not necessarily the most plausible model; it might make more sense for it to be able to decide at any time to go back to L1, but have to continue slowed development for time *delay* after the decision. The delay at the beginning model is a little easier to compute, and minimizes the L2d penalty, and thus is useful for computing bounds.) The age at L2 or L2d molt is 0, so that age at the L1 molt, which is parameter $a_{\min}$, is negative.

Computation of the hybrid option value proceeds in three steps, first specifying the basic parameters, then the delay and time, and finally the stock price. This works as follows:

vf = eav7ff[α, λ, σ, δ, dValue, l3Value, amin, qmax, r]
v = vf[a, delay]
$v[q]$ is the value.

a, q, α, λ, σ, δ, dValue, l3Value are as usual. $a_{\min}$ and delay were explained above. $q_{\max}$ and r are used in constructing the grid; reasonable defaults apply if these are not provided.

(Local) In[57]:=
```
eav7ff[α_?NumberQ, λ_?NumberQ, σ_?NumberQ, δ_?NumberQ, dValue_?NumberQ,
  l3Value_?NumberQ, amin_: Null, qmax_: Null, r_: Null, opts : OptionsPattern[]] :=
 Module[{aminv, waf, ugrid, agrid, umin, ϵ, n, h},
  aminv = If[amin === Null, -m`l2Stage, amin];
  {waf, agrid, ugrid, umin, ϵ, n, h} = wv7f[α, λ, σ, δ, dValue, l3Value, aminv];
  With[{amxv = aminv, wfv = waf, uminv = umin + ϵ, ugv = ugrid, ϵv = ϵ, ϰ0 = ϰ[0, α, λ, 0, δ]},
   Function[{a, delay},
    eav7iff[a, delay, amxv, dValue, l3Value, wfv, uminv, ugv, ϵv, α, λ, σ, δ, ϰ0]]
  ]
 ]
```

eav7iff and eav7if (not meant to be called directly by users) are the interface functions that do the dirty work of managing the interface at which early exercise is allowed. If $a \geq a_{\min}$ + delay, the option is still pure American, and everything is simple. But if $a_{\min} \leq a < a_{\min}$ + delay, it is to be evaluated as a European option whose terminal condition is given at $a_{\min}$ + delay by the American option value there. At that time, we have $v(a_{\min} + \text{delay}, 0) = V_d\, e^{\frac{\lambda}{\delta}(a_{\min}+\text{delay})}$ and $v(a_{\min} + \text{delay}, q) = V_{L3}\, e^{\lambda(a_{\min}+\text{delay})}\, q$ for large enough q. Also, for $a_{\min} + \text{delay} \leq a \leq 0$,

$$w_{\max}(a, u) = e^{-\frac{\lambda}{2\delta} a}\left(e^{\frac{\lambda}{\delta} a}\, e^{-u/2} + e^{\lambda a}\, e^{u/2}\right),$$

$$w(a, u) = w_{\max}(a, u) - w_1(a, u),$$

$$v(a, q) = e^{\frac{\lambda}{2\delta} a}\sqrt{V_d\, V_{L3}\, q}\;\; w_1(a, u),$$

$$v(a, q) = \left(e^{\frac{\lambda}{\delta} a}\, V_d + e^{\lambda a}\, V_{L3}\, q\right) - e^{\frac{\lambda}{2\delta} a}\sqrt{V_d\, V_{L3}\, q}\;\; w\!\left(a, \log\!\left(\frac{V_{L3}}{V_q}\, q\right)\right).$$

For $a_{\min} \leq a < a_{\min}$ + delay, I want to fix the decay rate,

$$\overline{w}_{\max}(a_{\min} + \text{delay}, u) = w_{\max}(a_{\min} + \text{delay}, u),$$

$$\overline{w}_{\max}(a_{\min} + \text{delay} - h, u) = e^{-\frac{\lambda}{2\delta} h}\, \overline{w}_{\max}(a_{\min} + \text{delay}, u)$$

Then I define $\overline{w}$ analogously,

$$\overline{w}(a, u) = \overline{w}_{\max}(a, u) - w_1(a, u)$$

w_1, $\overline{w}_{\max}$, and $\overline{w}$ are all solutions of the PDE, so I can get $\overline{w}(a_{\min} + \text{delay} - h, u)$ from $\overline{w}(a_{\min} + \text{delay}) = w(a_{\min} - \text{delay}, u)$ by convolution with the step for $-h$. Then

$$v(a, q) = e^{\frac{\lambda}{2\delta} a}\sqrt{V_d\, V_{L3}\, q}\;\; (\overline{w}_{\max}(a, u) - \overline{w}(a, u))$$

$$= e^{\frac{\lambda}{\delta} a}\left(V_d + e^{\left(\lambda - \frac{\lambda}{\delta}\right)(a_{\min}+\text{delay})}\, V_{L3}\, q\right) - e^{\frac{\lambda}{2\delta} a}\sqrt{V_d\, V_{L3}\, q}\;\; \overline{w}(a, u).$$

(Local) In[58]:=
```
eav7iff[a_, delay_, amin_, dValue_,
  l3Value_, waf_, umin_, ugrid_, ϵ_, α_, λ_, σ_, δ_, ϰ0_] :=
 Module[{we, whv, whf, wfa, umaxi, b},
  If[a ≥ amin + delay,
   (*
    * Pure American pricing
    *)
   umaxi = Min[-ugrid[[1]], ugrid[[-1]]];
   With[{wfv = waf, umax = umaxi, qmin = ⅇ^(-umaxi)},
    Function[q, av7if[a, q, dValue, l3Value, wfv, umax, qmin, ϰ0, λ]]
   ],
   (*
    * Hybrid European/American
    *)
   b = ⅇ^((λ - λ/δ) (amin+delay));
   we = waf[amin + delay, #] & /@ ugrid;
   whv = estep4[we, umin, amin + delay - a, ϵ, amin + delay, α, λ, σ, δ];
   whf = ListInterpolation[whv, {ugrid}];
   (* wfa=With[{wfv=wf},Function[{ignore,u},wfv[u]]]; *)
   With[{wfav = whf, b = b},
    Function[q, eav7if[a, q, amin, delay, λ, dValue, l3Value, wfav, umin, ϰ0, b]]
   ]
  ]
 ]
```

(Local) In[59]:=
```
eav7if[a_, q_, amin_, delay_, λ_, dValue_, l3Value_, waf_, umin_, ϰ0_, b_] :=
 Module[{u = If[q > 0, Log[(q l3Value)/dValue], -∞],
   qn = dValue/l3Value ⅇ^umin, qx = dValue/l3Value ⅇ^(-umin), df = ⅇ^(ϰ0 a), vf, qv},
  vf = Function[{qv, w}, df (df (dValue + b l3Value qv) - Sqrt[dValue l3Value qv] w)];
  Which[
   q < qn,
   Interpolation[{{qn, vf[qn, waf[umin]]}, {0, vf[0, 0]}}, q, InterpolationOrder → 1],
   q > qx, ⅇ^(λ (amin+delay)) ⅇ^(ϰ0 (a-amin-delay)) q,
   True, vf[q, waf[u]]
  ]
 ]
```

(Local) In[60]:=
```
ⅇ^((m`λ - m`λ/m`δ) (-m`l2Stage+m`delay))
```

(Local) Out[60]= 1.20483

(Local) In[61]:=
```
eav7if[-m`l2Stage, 1, -m`l2Stage, m`delay, λ, m`dValue,
  m`l3Value, 0 &, -10., ϰ[0, α, λ, 0, δ], ⅇ^((m`λ - m`λ/m`δ) (-m`l2Stage+m`delay))] // Simplify
```

(Local) Out[61]= $2.00285\, e^{-\frac{8.83922\,\lambda}{\delta}}$

(Local) In[62]:=
```
l2dv1[-m`l2Stage, Log[l3Value/dValue 1], α, λ, σ, δ, dValue, l3Value] /.
  {y → (0 &), dValue → m`dValue}
```

(Local) Out[62]= $0.512063\, e^{-\frac{8.83922\,\lambda}{\delta}}$

(Local) In[63]:=
```
l2v1[-m`l2Stage + m`delay, Log[m`l3Value/dValue 1], α, λ, σ, δ, dValue, m`l3Value]
```

(Local) Out[63]= $1.23734\, e^{-5.15621\,\lambda}$

(Local) In[64]:=
```
vff1 = eav7ff[m`α, m`λ, 0.001, m`δ, m`dValue, m`l3Value];
Short[vff1]
```

(Local) Out[65]//Short=
```
Function[{a$, delay$}, eav7iff[<<1>>]]
```

(Local) In[66]:=
```
vff2 = eav7ff[m`α, m`λ, 0.1, m`δ, m`dValue, m`l3Value];
Short[vff2]
```

(Local) Out[67]//Short=
```
Function[{a$, delay$}, eav7iff[<<1>>]]
```

(Local) In[68]:=
```
vff1[[2, 6]]
```

(Local) Out[68]=
```
InterpolatingFunction[{{-15.9844, 0.}, {-15.6001, 15.5924}}, <>]
```

(Local) In[69]:=
```
Plot[vff1[[2, 6]][-m`l2Stage + m`delay, u], {u, -15, 15}]
```

(Local) Out[69]=

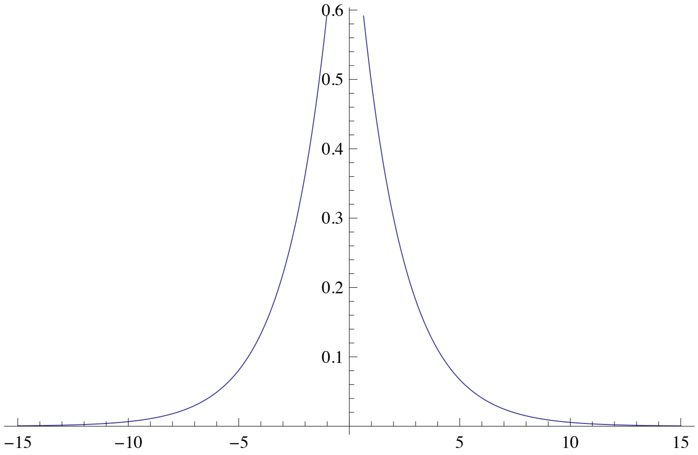

(Local) In[70]:=
```
vf1 = vff1[-m`l2Stage, m`delay]
```

(Local) Out[70]=
```
Function[q$, eav7if[-8.83922, q$, -8.83922, 3.68301, 0.0421625, 0.512063, 1.23734,
  InterpolatingFunction[{{-15.6001, 15.5924}}, <>], -15.5924, 0.0391509, 1.20483]]
```

(Local) In[71]:=
```
vf2 = vff2[-m`l2Stage, m`delay]
```

(Local) Out[71]=
```
Function[q$, eav7if[-8.83922, q$, -8.83922, 3.68301, 0.0421625, 0.512063, 1.23734,
  InterpolatingFunction[{{-15.6001, 15.5924}}, <>], -15.5924, 0.0391509, 1.20483]]
```

(Local) In[72]:=
```
{vf1[1], vf2[1]}
```

(Local) Out[72]=
```
{0.746155, 0.780043}
```

(Local) In[73]:=
```
With[{a = -m`l2Stage, α = m`α, λ = m`λ,
   σ = m`σ, δ = m`δ, dValue = m`dValue, l3Value = m`l3Value},
  Plot[{
    vf1[q], vf2[q],
    l2div2[a, q, α, λ, σ, δ, dValue, l3Value]
   }, {q, 0, 1},
   PlotStyle → {Red, Green, Black}]
 ]
```

(Local) Out[73]=

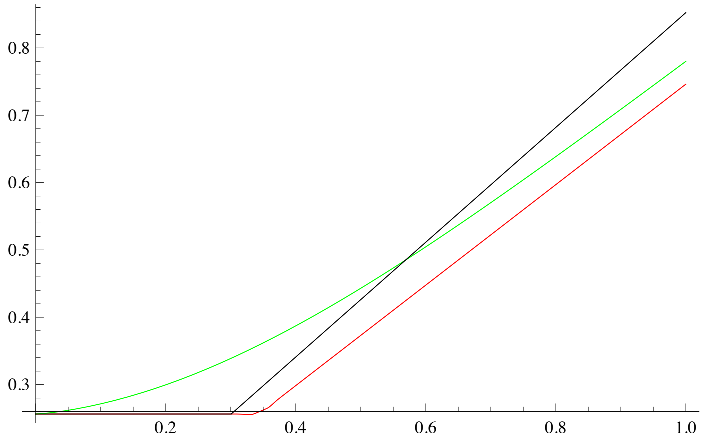

Now it makes sense that the value is less than intrinsic value, because the worm has to grow two hours at the slow L2d rate before being able to exercise.

Maximum and minimum value

minValue[q, a, α, λ, δ, delay, V_d, V_{L3}] is the value of the L2d at volatility zero.

(Local) In[74]:=
```
minValue[q_, a_: -m`l2Stage, α_: m`α, λ_: m`λ, δ_: m`δ,
   amin_: -m`l2Stage, delay_: m`delay, dValue_: m`dValue, l3Value_: m`l3Value] :=
  If[a ≥ amin + delay,
   Max[dValue e^(λ/δ a), l3Value q e^(λ a)],
   Max[dValue e^(λ/δ a), l3Value q e^(-λ/δ delay+λ(a+delay))]
  ]
```

minValue[q, a, α, λ, δ, delay, V_d, V_{L3}] is the value of the L2d at infinite volatility.

(Local) In[75]:=
```
maxValue[q_, a_: -m`l2Stage, α_: m`α, λ_: m`λ, δ_: m`δ,
   amin_: -m`l2Stage, delay_: m`delay, dValue_: m`dValue, l3Value_: m`l3Value] :=
  If[a ≥ amin + delay,
   dValue e^(λ/δ a) + l3Value q e^(λ a),
   dValue e^(λ/δ a) + l3Value q e^(-λ/δ delay+λ(a+delay))
  ]
```

(Local) In[76]:= **Plot**$\left[\left\{\text{minValue}[q],\ \text{maxValue}[q],\ e^{-\text{m`}\lambda\ \text{m`l2Stage}}\ \text{m`l3Value}\ q\right\},\ \{q,\ 0,\ 3\}\right]$

(Local) Out[76]=

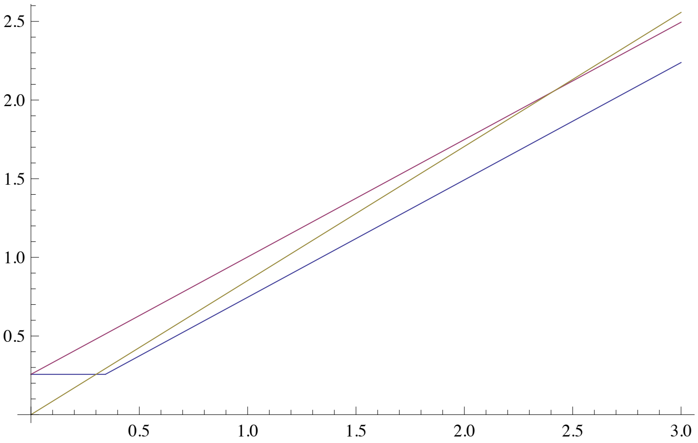

Minimum and maximum cutoff

The value of the L2 is $V_{L3}\, q e^{\lambda a}$. At zero volatility, the value of the L2d is $\max\left(V_d\, e^{\frac{\lambda}{\delta} a},\ V_{L3}\, q e^{-\frac{\lambda}{\delta}\text{delay}+\lambda(a+\text{delay})}\right)$. Only can intersect the L2 value, so I get

(Local) In[77]:= **Solve**$\left[\text{dValue}\ e^{\frac{\lambda}{\delta} a} == \text{l3Value}\ q\ e^{\lambda a},\ q\right][[1]]$ // **Simplify**

(Local) Out[77]= $\left\{q \to \frac{\text{dValue}\ e^{a\left(-1+\frac{1}{\delta}\right)\lambda}}{\text{l3Value}}\right\}$

(Local) In[78]:= **minCutoff**[a_: - m`l2Stage, α_: m`α, λ_: m`λ, δ_: m`δ,
delay_: m`delay, dValue_: m`dValue, l3Value_: m`l3Value] := **Evaluate**[
q /. **Solve**$\left[\text{dValue}\ e^{\frac{\lambda}{\delta} a} == \text{l3Value}\ q\ e^{\lambda a},\ q\right][[1]]$ // **Simplify**
];
{**minCutoff**[a, α, λ, δ, delay, dValue, l3Value], **minCutoff**[]}

(Local) Out[79]= $\left\{\frac{\text{dValue}\ e^{a\left(-1+\frac{1}{\delta}\right)\lambda}}{\text{l3Value}},\ 0.300679\right\}$

At infinite volatility, the value of the L2d is $V_d\, e^{\frac{\lambda}{\delta} a} + V_{L3}\, q e^{-\frac{\lambda}{\delta}\text{delay}+\lambda(a+\text{delay})}$.

(Local) In[80]:= **Solve**$\left[\text{dValue}\ e^{\frac{\lambda}{\delta} a} + \text{l3Value}\ q\ e^{-\frac{\lambda}{\delta}\text{delay}+\lambda(\text{a}+\text{delay})} == \text{l3Value}\ q\ e^{\lambda a},\ q\right][[1]]$ // **Simplify**

(Local) Out[80]= $\left\{q \to \frac{\text{dValue}\ e^{\frac{(\text{a}+\text{delay})\lambda}{\delta}}}{\left(-e^{(\text{a}+\text{delay})\lambda} + e^{\left(\text{a}+\frac{\text{delay}}{\delta}\right)\lambda}\right)\text{l3Value}}\right\}$

(Local) In[81]:=
```
maxCutoff[a_: -m`l2Stage, α_: m`α, λ_: m`λ, δ_: m`δ,
   delay_: m`delay, dValue_: m`dValue, l3Value_: m`l3Value] := Evaluate[
   q /. Solve[dValue ⅇ^(λ/δ a) + l3Value q ⅇ^(-λ/δ delay+λ(a+delay)) == l3Value q ⅇ^(λa), q][[1]] // Simplify
  ];
{maxCutoff[a, α, λ, δ, delay, dValue, l3Value], maxCutoff[]}
```

(Local) Out[82]=

$$\left\{\frac{\text{dValue}\, e^{\frac{(a+\text{delay})\,\lambda}{\delta}}}{\left(-e^{(a+\text{delay})\,\lambda}+e^{\left(a+\frac{\text{delay}}{\delta}\right)\lambda}\right)\text{l3Value}},\ 2.4127\right\}$$

Gadget

(Local) In[83]:=
```
Module[{eavff, evf, avf, ep, ap, σv = Null, delayv = Null, av = Null,
   a, σ, delay, α = m`α, λ = m`λ, dValue = m`dauerValue, l3Value = m`l3Value,
   δ = m`δ, amin = -m`l2Stage, amax = m`l2Stage, qmax = 2, vmax = 3, ar = 1, qp},
  qp = Plot[Evaluate[l3Value {
        q
       }],
    {q, 0, qmax}, PlotRange → {0, vmax},
    AspectRatio → ar,
    PlotStyle → {Green}];
  Manipulate[
   Module[{eavf, eap},
    If[σ =!= σv,
     eavff = eav7ff[α, λ, σ, δ, dValue, l3Value];
     evf = eavff[a, amax];
     avf = eavff[a, 0];
     ep = Plot[Evaluate[l3Value {
           evf[q] / l2div2[a, 1, α, λ, σ, δ, dValue, l3Value]
          }],
        {q, 0, qmax}, PlotRange → {0, vmax},
        AspectRatio → ar,
        PlotStyle → {Directive[Thick, Red]}];
     ap = Plot[Evaluate[l3Value {
           avf[q] / l2div2[a, 1, α, λ, σ, δ, dValue, l3Value]
          }],
        {q, 0, qmax}, PlotRange → {0, vmax},
        AspectRatio → ar,
        PlotStyle → {Directive[Thick, Black]}];
     σv = σ;
    ];
    If[a =!= av,
     evf = eavff[a, amax];
     avf = eavff[a, 0];
     ep = Plot[Evaluate[l3Value {
           evf[q] / l2div2[a, 1, α, λ, σ, δ, dValue, l3Value]
          }],
        {q, 0, qmax}, PlotRange → {0, vmax},
        AspectRatio → ar,
        PlotStyle → {Directive[Thick, Red]}];
     ap = Plot[Evaluate[l3Value {
           avf[q] / l2div2[a, 1, α, λ, σ, δ, dValue, l3Value]
          }],
        {q, 0, qmax}, PlotRange → {0, vmax},
        AspectRatio → ar,
```

```
      PlotStyle → {Directive[Thick, Black]}];
    av = a;
   ];
   eavf = eavff[a, delay];
   eap = Plot[Evaluate[l3Value {
         eavf[q] / l2div2[a, 1, α, λ, σ, δ, dValue, l3Value]
       }],
     {q, 0, qmax}, PlotRange → {0, vmax},
     AspectRatio → ar,
     PlotStyle → {Directive[Thick, Blue]}];
   Show[ep, ap, eap, qp]
  ],
  {{a, amin, "a"}, amin, 0, Appearance → "Labeled"},
  {{σ, 0.1, "σ"}, 0, 1, Appearance → "Labeled", ContinuousAction → False},
  {{delay, m`delay, "delay"}, 0, amax, Appearance → "Labeled"}
 ]
]
```

(Local) Out[83]=

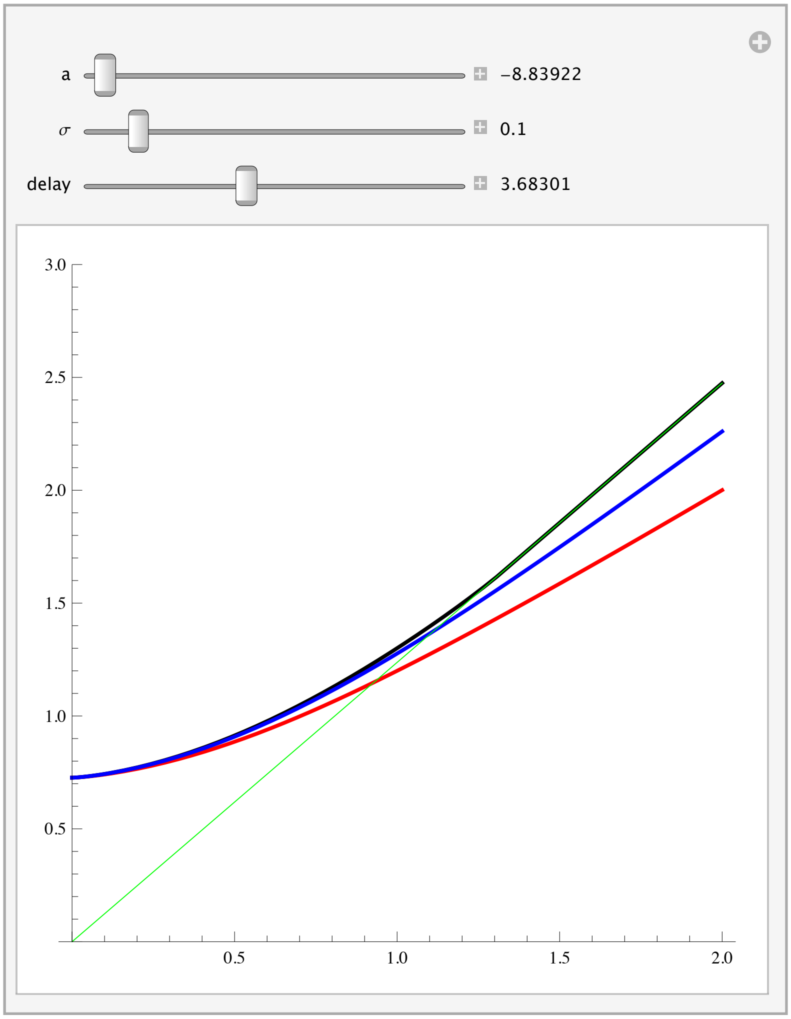

Korean flag plot

(Local) In[84]:=
```
acutoff[a_, α_?NumberQ, λ_?NumberQ, σ_?NumberQ, δ_?NumberQ,
  delay_?NumberQ, dValue_?NumberQ, l3Value_?NumberQ, qmax_: Null, r_: Null] :=
 Module[{l2dvff, l2dvf, l2vf, q},
  l2dvff = eav7ff[α, λ, σ, δ, dValue, l3Value, a, qmax, r];
  l2dvf = l2dvff[a, delay];
  l2vf = Function[q, ⅇ^(λ a) l3Value q];
  q /. FindRoot[l2vf[q] == l2dvf[q], {q, dValue}]
 ]
```

(Local) In[85]:=
```
Quiet[
 acutoff[-m`l2Stage, m`α, m`λhat, #, m`δ, m`delay, m`dValue, m`l3Value] & /@
  {0.0001, 0.001, 0.1, 0.3, 1, 10},
 InterpolatingFunction::"dmval"
]
```

(Local) Out[85]=
```
{0.300679, 0.300677, 0.568805, 1.38082, 2.30963, 2.4127}
```

(Local) In[86]:=
```
{minCutoff[], maxCutoff[]}
```

(Local) Out[86]=
```
{0.300679, 2.4127}
```

Hurray!

This will be slow...

(Local) In[87]:=
```
Quiet[
 Timing[acf1 = FunctionInterpolation[acutoff[-m`l2Stage,
      m`α, m`λhat, σ, m`δ, m`delay, m`dValue, m`l3Value], {σ, 0.001, 10},
    PrecisionGoal → ∞, AccuracyGoal → 4,
    MaxRecursion → 6]
 ],
 {FindRoot::"lstol", InterpolatingFunction::"dmval", FunctionInterpolation::ncvb}]
```

(Local) Out[87]=
```
{520.086228, InterpolatingFunction[{{0.001, 10.}}, <>]}
```

(Local) In[88]:=
```
Module[
 {σmin = 0.01, σmax = 1., vmin = minCutoff[], vmid, vmax = maxCutoff[],
  σ, σmid, lσleft, lσright, σleft, σright, vleft, vright, diag},
 (* vmid=(vmin+vmax)/2; *)
 diag[σ_] := Interpolation[{{σmin, vmax}, {σmax, vmin}}, σ, InterpolationOrder → 1];
 σmid = σ /. FindRoot[acf1[σ] == diag[σ], {σ, (σmin + σmax)/2}]; (* lσleft=(Log[σmin]+Log[σmid])/2; *)
 (* lσright=(Log[σmid]+Log[σmax])/2; *)
 σleft = (σmin + σmid)/2;
 σright = (σmid + σmax)/2; (* {vleft,vright}=acf1[ⅇ^#]&/@{lσleft,lσright}; *)
 {vleft, vright} = diag /@ {σleft, σright};
 Quiet[
  Plot[{acf1[σ], acf1[σ]}, {σ, σmin, σmax},
   PlotRange → {vmin, vmax},
   PlotStyle → Directive[Transparent],
   Filling → {1 → {Bottom, Blue}, 2 → {Top, Red}},
   Epilog → {
     Style[Text["L2", {σleft, vleft}], 48, Bold, Black, FontFamily → "Helvetica"],
     Style[Text["L2d", {σright, vright}], 48, Bold, Yellow, FontFamily → "Helvetica"]
    },
   ImageSize → 400,
   AspectRatio → 1,
   Frame → True,
   FrameLabel → {
     Style["volatility", Bold, Medium, FontFamily → "Helvetica"],
     Style["quality", Bold, Medium, FontFamily → "Helvetica"]}],
  InterpolatingFunction::"dmval"]
]
```

(Local) Out[88]=

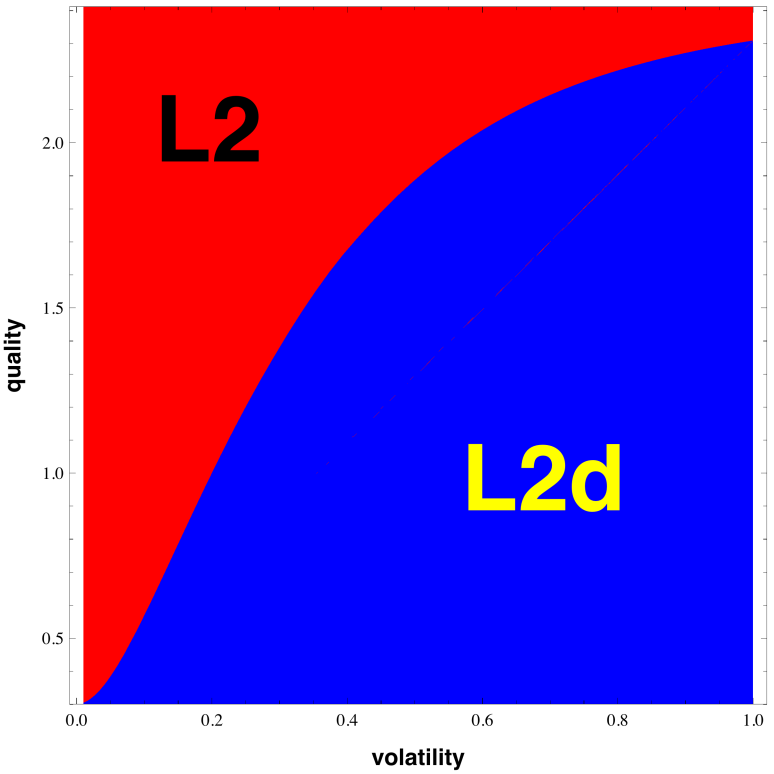

With $\alpha = 0.057$, the modal L2 takes 9.6 h to reach the molt, and the L2d 32.8 h. A volatility of 0.2 therefore means that the SD of $\log q$ is 0.59, which corresponds to a factor of 1.8, which is quite plausible. Suppose I said the maximum plausible $\sigma_{\log q} = 10^6$. That would be a volatility of $\sigma = 4.65$, which is pretty close to saturating:

(Local) In[89]:=
```
acf1 /@ {0.01, 0.2, 0.6486926092715758`, 10}
```

(Local) Out[89]=
```
{0.304703, 1.00099, 2.0952, 2.4127}
```

(Local) In[90]:=
$$\frac{\texttt{acf1[0.01] + acf1[10]}}{2}$$

(Local) Out[90]=
```
1.3587
```

(Local) In[91]:=
$$\texttt{FindRoot}\left[\texttt{acf1}[\sigma] == \frac{\texttt{acf1[0.01] + acf1[10]}}{2}, \{\sigma, 0.2\}\right]$$

(Local) Out[91]=
```
{σ → 0.293448}
```

Here's the same function, but plotted against $e^{\sigma^2 T}$.

(Local) In[92]:=
$$\texttt{Simplify}\left[\texttt{Solve}\left[x == e^{\sigma\sqrt{T}}, \sigma, \texttt{Reals}\right], T > 0 \;\&\&\; x > 0\right]$$

(Local) Out[92]=
$$\left\{\left\{\sigma \to \frac{\texttt{Log}[x]}{\sqrt{T}}\right\}\right\}$$

(Local) In[93]:=
```
m`α
```

(Local) Out[93]=
```
0.0569294
```

(Local) In[94]:=
```
Module[
 {σmin = 0.01, σmax, vmin = Floor[minCutoff[] - 0.05, 0.1],
  vmid, vmax = Ceiling[maxCutoff[] + 0.05, 0.1], T = 2 m`l2dStage,
  lx, x, xmin, xmid, xmax = 1.0 × 10.^3, xleft, xright, σ, σmid,
  lσleft, lσright, σleft, σright, vleft, vright, diag, σ2x, x2σ},
 σ2x[σ_] := e^(σ √T) - 1;
 x2σ[x_] := Log[x + 1] / √T;
 σmax = x2σ[xmax];
 (* vmid= (vmin+vmax)/2; *)
 diag[lx_] :=
  Interpolation[{{Log[xmin], vmax}, {Log[xmax], vmin}}, lx, InterpolationOrder → 1];
 xmin = σ2x[σmin];
 xmid = e^lx /. FindRoot[acf1[x2σ[e^lx]] == diag[lx], {lx, Log[√(xmin xmax)]}];
 (* lσleft= (Log[σmin]+Log[σmid])/2; *)
 (* lσright= (Log[σmid]+Log[σmax])/2; *)
 xleft = √(xmin xmid);
 xright = √(xmid xmax); (* {vleft,vright}=acf1[e^#]&/@{lσleft,lσright}; *)
 {vleft, vright} = diag /@ {Log[xleft], Log[xright]};
 Quiet[
  LogLinearPlot[{acf1[x2σ[x]], acf1[x2σ[x]]}, {x, xmin, xmax},
   PlotRange → {vmin, vmax},
   PlotStyle → Directive[Transparent],
   Filling → {1 → {Bottom, Blue}, 2 → {Top, Red}},
   Epilog → {
     Style[Text["L2", {Log[xleft], vleft}], 48, Bold, Black, FontFamily → "Helvetica"],
     Style[Text["L2d", {Log[xright], vright}],
      48, Bold, Yellow, FontFamily → "Helvetica"]
    },
   ImageSize → 400,
   AspectRatio → 1,
   Frame → True,
   FrameStyle → Directive[Bold, Black, FontSize → 12, FontFamily → "Helvetica"],
   FrameLabel → {
     Style["uncertainty", Bold, Medium, FontFamily → "Helvetica"]
     (* uncertainty = exp(σ√(T/α))-1 *),
     Style["predicted environment quality", Bold, Medium, FontFamily → "Helvetica"]}],
  InterpolatingFunction::"dmval"]
]
```

(Local) Out[94]=

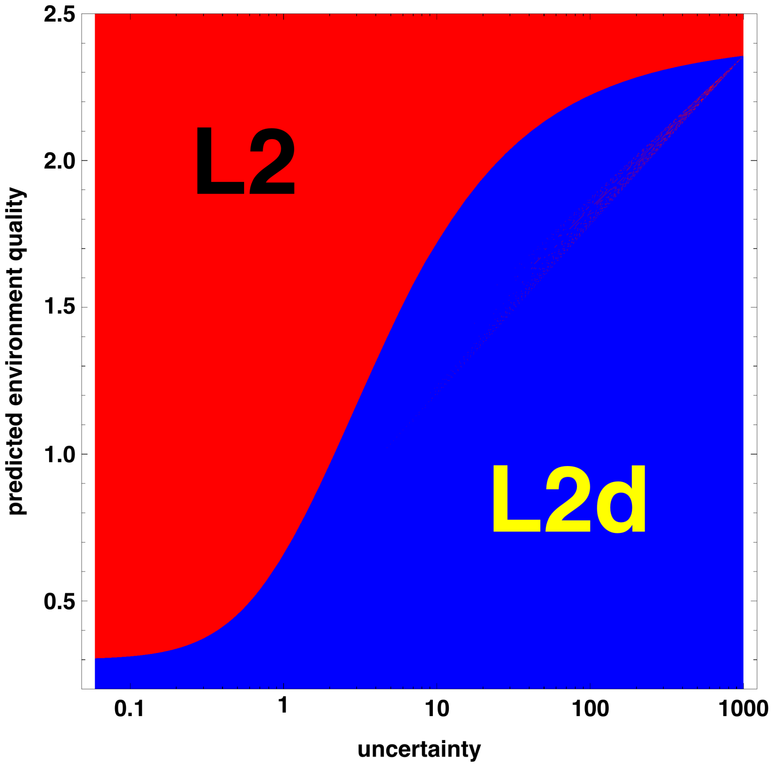
2.5
2.0
1.5
1.0
0.5
predicted environment quality
L2
L2d
0.1
1
10
100
1000
uncertainty

Value plot

(Local) In[95]:=
```
Module[{q, σ, T = 2 m`l2dStage, eσsTs = {0.5, 2}, σs,
   is = 400, pfs, ps, lfs = 10, qmax = 1, vmax = 1, ar = 1, vffs, vfs,
   dLabelPos, l2LabelPos, u1LabelPos, uiLabelPos, uLabelPoss,
   α = m`α, λ = m`λ, δ = m`δ, delay = m`delay,
   dValue = m`dValue, l3Value = m`l3Value, amin = -m`l2Stage},
  σs = Log[eσsTs + 1] / √T ;
  vffs = eav7ff[α, λ, #, δ, dValue, l3Value, amin] & /@ σs;
  vfs = #[amin, delay][q] & /@ vffs;
  dLabelPos = {0.75 qmax, ⅇ^(λ/δ amin) dValue};
  l2LabelPos = With[{q = 0.15 qmax}, {q, ⅇ^(λ amin) l3Value q}];
  u1LabelPos =
   With[{q = 0.5 qmax}, {q, minValue[q, amin, α, λ, δ, amin, delay, dValue, l3Value]}];
  uiLabelPos = With[{q = 0.4 qmax},
    {q, maxValue[q, amin, α, λ, δ, amin, delay, dValue, l3Value]}];
  uLabelPoss = With[{q = 0.4 qmax},
    {q, #[amin, delay][q]} & /@ vffs
   ];
  pfs = { (* functions to plot *)
     ⅇ^(λ amin) l3Value q, (* L2 *)
     ⅇ^(λ/δ amin) dValue, (* dauer-commited L2d *)
     minValue[q, amin, α, λ, δ, amin, delay, dValue, l3Value], (* 0 volatility *)
     maxValue[q, amin, α, λ, δ, amin, delay, dValue, l3Value] (* infinite volatility *)
    }~Join~
    vfs;
  ps = { (* PlotStyles *)
     Directive[Darker[Green, 0.5], AbsoluteThickness[2]],
     Directive[Gray, AbsoluteThickness[5], Dashed],
     Directive[Black, AbsoluteThickness[2]],
     Directive[Black, AbsoluteThickness[2]]}~Join~
    ConstantArray[Directive[Black, AbsoluteThickness[1]], Length[σs]];
  Plot[
   Evaluate[pfs], {q, 0, qmax},
   PlotStyle → ps,
   Epilog → {
      Style[Text["dauer", dLabelPos, {0, 1}],
       Bold, Gray, FontSize → 12, FontFamily → "Helvetica"],
      Style[Text["L2", l2LabelPos, {0, -1}], Bold, Darker[Green, 0.5],
       FontSize → 12, FontFamily → "Helvetica"],
      Style[Text["0", u1LabelPos, {-1, 1}], Bold, Black,
       FontSize → lfs, FontFamily → "Helvetica"],
      Style[Text["∞", uiLabelPos, {1, -1}], Bold, Black,
       FontSize → lfs, FontFamily → "Helvetica"]
     }~Join~
     (Style[Text["" <> ToString[#1], #2, {1, -1}], Bold, Black, FontSize → lfs,
         FontFamily → "Helvetica"] & @@@ Transpose[{eσsTs, uLabelPoss}]),
   Frame → {{True, False}, {True, False}},
   FrameStyle → Directive[Bold, Black, FontSize → 12, FontFamily → "Helvetica"],
   FrameLabel → {"predicted environment quality", "value (dauers)"},
   PlotRange → {{0, qmax}, {0, vmax}},
   ImageSize → is, AspectRatio → ar
  ]
 ]
```

(Local) Out[95]=

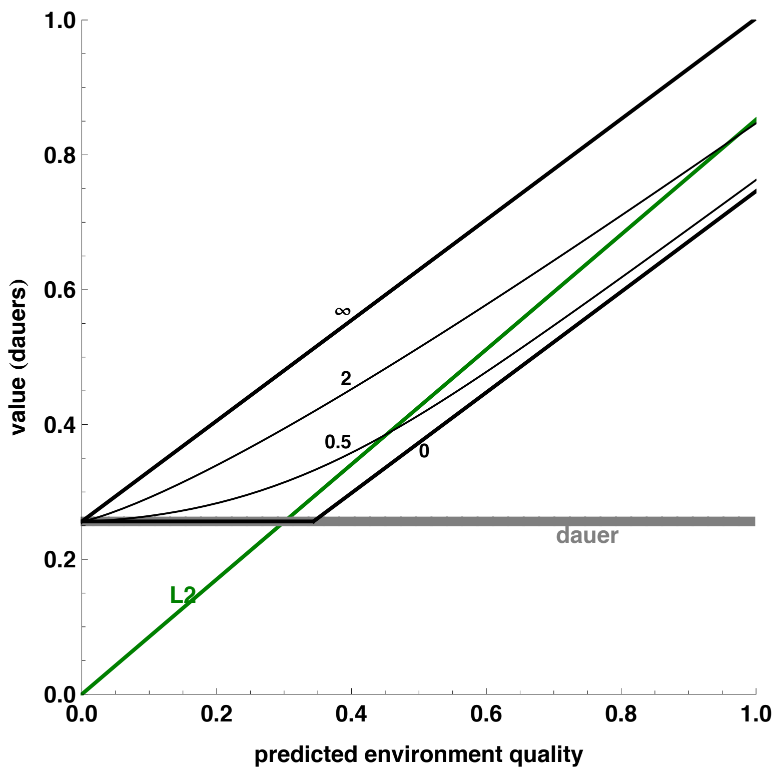

Here's where the intersections are in the above plot:

(Local) In[96]:=
```
{minCutoff[]} ~ Join ~
  With[{T = 2 m`l2dStage}, acf1[Log[# + 1] / Sqrt[T]] & /@ {0.5, 2}] ~ Join ~ {maxCutoff[]}
```

(Local) Out[96]=
```
{0.300679, 0.453832, 0.966183, 2.4127}
```

Volatility-free decision-making

Suppose you live in a world where the volatility is either 0 or ∞ with equal probability. You are going to make the L2/L2d decision purely on the basis of environment quality, ignoring volatility. What is the optimal q boundary, and how much value do you sacrifice compared to an optimal choice? This is a little tricky, because there are actually two decision points: the L1 molt, and the early exercise point within the L2d.

Let’s consider first the smart worm, the worm the model has been based on. This worm knows both the environment and the volatility and makes decisions based on both. First, suppose the volaility is 0. The worm knows at the time of the L1 molt what the environment is going to look like forever, so it will choose L2d only if it eventually wants to go dauer, and it will choose L2 only if it eventually wants to go L3. Its value is therefore

(Local) In[97]:=
```
$Assumptions = Union[$Assumptions && delay > 0 && amin < 0]
```

(Local) Out[97]=
```
u ∈ Reals && delay > 0 && dValue > 0 && l3Value > 0 &&
  α > 0 && λ > 0 && ν > 0 && σ > 0 && amin < 0 && 0 < δ < 1 && 0 ≤ t ≤ T
```

(Local) In[98]:=
```
v0sv1[q_, a_: -m`l2Stage, α_: m`α, λ_: m`λ, δ_: m`δ, amin_: -m`l2Stage,
    delay_: m`delay, dValue_: m`dValue, l3Value_: m`l3Value] :=
  Max[e^(λ/δ a) dValue, e^(λ a) l3Value q];
v0sv1[q, amin, α, λ, δ, amin, delay, dValue, l3Value]
```

(Local) Out[99]=

$$\text{Max}\left[\text{dValue}\, e^{\frac{\text{amin}\,\lambda}{\delta}},\ e^{\text{amin}\,\lambda}\, \text{l3Value}\, q\right]$$

In the infinite volatility world, there are three options to consider. First, you may choose L2. That's simple: you have value $e^{\text{amin}\,\lambda}$ l3Value q. Second, you may choose L2d and remain L2d all the way to the molt, then become dauer. Second, you may choose L2d, remain L2d until the early exercise point, then switch to L2. At infinite volatility you never need to switch to the L2 after the early exercise point, because the environment will either have become very good or very bad by a_{EE}. It is good with probability that approaches 0 but with quality that approaches infinity, and bad with probability that approaches 1 and quality that approaches 0, in such a way that the mean is q. I've already worked out that the value in this case is the sum, maxValue.

(Local) In[100]:=
```
vinfsv1[q_, a_: -m`l2Stage, α_: m`α, λ_: m`λ, δ_: m`δ, amin_: -m`l2Stage,
    delay_: m`delay, dValue_: m`dValue, l3Value_: m`l3Value] :=
  Max[maxValue[q, a, α, λ, δ, amin, delay, dValue, l3Value], e^(λ a) l3Value q];
vinfsv1[q, amin, α, λ, δ, amin, delay, dValue, l3Value] // Simplify
```

(Local) Out[101]= $\text{Max}\left[e^{\text{amin}\,\lambda}\,\text{l3Value}\,q,\ \text{dValue}\,e^{\frac{\text{amin}\,\lambda}{\delta}} + e^{\left(\text{amin}+\text{delay}-\frac{\text{delay}}{\delta}\right)\lambda}\,\text{l3Value}\,q\right]$

In the infinite volatility world, the L2/L2d cutoff is

(Local) In[102]:=
```
Solve[
  Equal @@ (vinfsv1[q, amin, α, λ, δ, amin, delay, dValue, l3Value] // Simplify),
  q
 ][[1, 1]]
```

(Local) Out[102]= $q \to \frac{\text{dValue}\,e^{\frac{\text{amin}\,\lambda}{\delta}}}{\left(e^{\text{amin}\,\lambda} - e^{\left(\text{amin}+\text{delay}-\frac{\text{delay}}{\delta}\right)\lambda}\right)\text{l3Value}}$

(Local) In[103]:=
```
vinfqco1 = q /. Solve[
    Equal @@ (vinfsv1[q] // Simplify),
    q
   ][[1, 1]]
```

(Local) Out[103]= 2.4127

The average value of the smart worm is then

(Local) In[104]:=
```
vsv1[q_, a_: -m`l2Stage, α_: m`α, λ_: m`λ, δ_: m`δ, amin_: -m`l2Stage,
    delay_: m`delay, dValue_: m`dValue, l3Value_: m`l3Value] :=
  1/2 (v0sv1[q, a, α, λ, δ, amin, delay, dValue, l3Value] +
     vinfsv1[q, a, α, λ, δ, amin, delay, dValue, l3Value]);
{
  vsv1[q, amin, α, λ, δ, amin, delay, dValue, l3Value],
  vsv1[q]
 } // Simplify
```

(Local) Out[105]= $\left\{\frac{1}{2}\left(\text{Max}\left[\text{dValue}\,e^{\frac{\text{amin}\,\lambda}{\delta}},\ e^{\text{amin}\,\lambda}\,\text{l3Value}\,q\right] + \text{Max}\left[e^{\text{amin}\,\lambda}\,\text{l3Value}\,q,\ \text{dValue}\,e^{\frac{\text{amin}\,\lambda}{\delta}} + e^{\left(\text{amin}+\text{delay}-\frac{\text{delay}}{\delta}\right)\lambda}\,\text{l3Value}\,q\right]\right),\right.$
$\left.0.5\,(\text{Max}[0.256293,\ 0.852381\,q] + \text{Max}[0.256293 + 0.746155\,q,\ 0.852381\,q])\right\}$

(Local) In[106]:=
```
PiecewiseExpand[vsv1[q]] // Simplify
```

(Local) Out[106]= $\begin{cases} 0.852381\,q & q > 2.4127 \\ 0.128146 + 0.799268\,q & 0.300679 < q \le 2.4127 \\ 0.256293 + 0.373077\,q & \text{True} \end{cases}$

(Local) In[107]:= $\left\{\text{v0sv1}[q],\ e^{-\frac{\text{m`}\lambda}{\text{m`}\delta}\,\text{m`l2Stage}}\,\text{m`dValue},\ e^{-\text{m`}\lambda\,\text{m`l2Stage}}\,\text{m`l3Value}\,q,\ \text{vinfsv1}[q]\right\}$

(Local) Out[107]= {Max[0.256293, 0.852381 q], 0.256293, 0.852381 q, Max[0.256293 + 0.746155 q, 0.852381 q]}

(Local) In[108]:=
```
Plot[{
   E^(-(m`λ/m`δ) m`l2Stage) m`dValue, E^(-m`λ m`l2Stage) m`l3Value q,
   vsv1[q], v0sv1[q], vinfsv1[q]
  },
  {q, 0, 3},
  PlotStyle → {Gray, Gray, Directive[Thick, Black], Blue, Red},
  PlotRange → {Automatic, {0, Automatic}}
 ]
```

(Local) Out[108]=

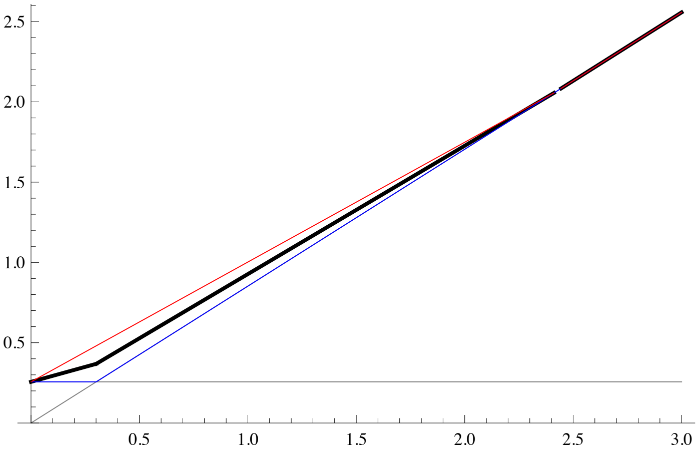

So, there are two breakpoints. (This is especially clear from the PiecewiseExpand result.) The first, at 0.3, is the boundary below which it is always best to go L2d, even in a zero volatility world. The second, at 2.4, is the boundary above which it is always better to go L2, even in any infinitely volatile world, because the option to go dauer if things turn sour is not worth even the minimal L2d delay.

OK, now for the dumb worm, which doesn't know the volatility. Consider first the zero volaility case. There is a possibility here that didn't occur with the smart worm: early exercise can happen. If I'm an L2d at a_{EE} and I progress to dauer, my value is $e^{\frac{\lambda}{\delta} a_{\text{EE}}} V_{\text{dauer}}$. If I switch to L2, my value is $e^{\lambda a_{\text{EE}}} V_{\text{L3}}\, q$. Is it safe to assume early exercise always occurs at a_{EE}? Well, intuitively, yes. If the volaility is 0, q will not change, so there is no point in waiting—you just lose value because of delay. If the volatility is infinite then q is almost surely very large or 0 at a_{EE}, so you should choose right away. So, in the zero volatility case the dumb L2d's value at a_{EE} is $\max\left(e^{\frac{\lambda}{\delta} a_{\text{EE}}} V_{\text{dauer}}, e^{\lambda a_{\text{EE}}} V_{\text{L3}}\, q\right)$. At the L1 molt, this is discounted further by $e^{-\frac{\lambda}{\delta}\text{delay}}$, so the worm's value is just minValue. In the high volatility world, the L2d has value maxValue. Since the worm doesn't know which world it lives in, the L2d's value at the L1 molt is just the average:

(Local) In[109]:=
```
vl2ddv1[q_, a_: -m`l2Stage, α_: m`α, λ_: m`λ, δ_: m`δ, amin_: -m`l2Stage,
   delay_: m`delay, dValue_: m`dValue, l3Value_: m`l3Value] :=
  1/2 (minValue[q, a, α, λ, δ, amin, delay, dValue, l3Value] +
     maxValue[q, a, α, λ, δ, amin, delay, dValue, l3Value]);
{vl2ddv1[q, amin, α, λ, δ, amin, delay, dValue, l3Value],
  vl2ddv1[q]} // Simplify
```

(Local) Out[110]=

$$\left\{\frac{1}{2}\left(\text{dValue}\, e^{\frac{\text{amin}\,\lambda}{\delta}} + e^{\left(\text{amin}+\text{delay}-\frac{\text{delay}}{\delta}\right)\lambda}\, \text{l3Value}\, q + \text{Max}\left[\text{dValue}\, e^{\frac{\text{amin}\,\lambda}{\delta}},\, e^{\left(\text{amin}+\text{delay}-\frac{\text{delay}}{\delta}\right)\lambda}\, \text{l3Value}\, q\right]\right),\right.$$
$$\left.\frac{1}{2}\left(0.256293 + 0.746155\, q + \text{Max}[0.256293,\, 0.746155\, q]\right)\right\}$$

(Local) In[111]:=
```
PiecewiseExpand[vl2ddv1[q]] // Simplify
```

(Local) Out[111]=
```
{ 0.128146 + 0.746155 q   q > 0.343485
  0.256293 + 0.373077 q   True
```

This has the same bilinear kinked shape as the value curve for the smart worm, but interestingly, with the kink in a different place:

(Local) In[112]:=
```
Plot[{
  ⅇ^(-(m`λ/m`δ) m`l2Stage) m`dValue, ⅇ^(-m`λ m`l2Stage) m`l3Value q, ⅇ^((-m`l2Stage+m`delay-m`delay/m`δ) m`λ) m`l3Value q,
  v0sv1[q], vsv1[q], minValue[q], vl2ddv1[q]
 },
 {q, 0, 0.5},
 PlotStyle → {Gray, Gray, Gray, Blue, Blue, Red, Red},
 PlotRange → {Automatic, {0, Automatic}}
]
```

(Local) Out[112]=

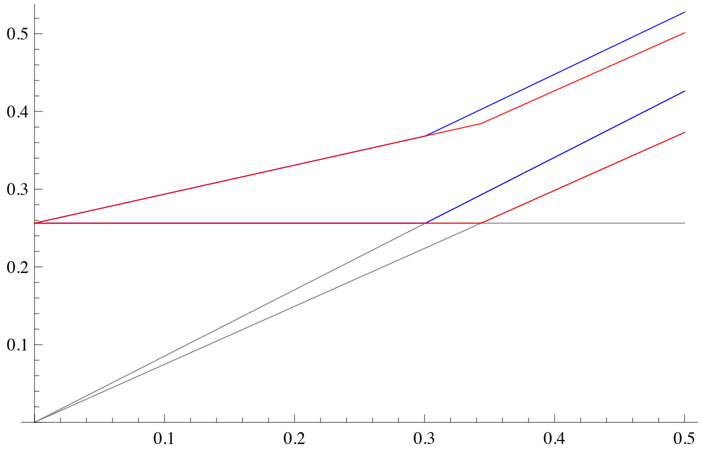

OK, this makes it clear. The break in the smart worm curve is inherited from the zero-volaility world, and it occurs where the worm committed to L2's value meets that of a hypothetical worm committed to dauer. In this world the L2d option is worthless and is never used. But the dumb worm doesn't know what world it's in, so it always has to budget for the possibility of volatility, which gives the option value. Since volatility, if nonzero, is infinite, the option, if exercised, will always be exercised at the earliest possible time. So this breakpoint occurs at the line for an L3 discounted by the time that a worm who goes L2d then switches to L2 spends.

Behaviorally, what is going on in this region between 0.3 and 0.34? The dumb worm is making a suboptimal decision in the zero volatility world by choosing L2d, but this is true all the way up to 1.2, the cutoff I identify below for the dumb worm's decision. The difference is that, below 0.34, early exercise doesn't occur. When the worm reaches the early exercise point it has already paid part of the price for becoming a dauer, and its best strategy now is to suck it up and go on to become a dauer. Above 0.34, however, it abandons the L2d pathway and switches to L2, thus recouping most of the loss caused by the earlier error. It is in a sneaky way using volatility information, since q would be almost surely outside the range [0.34, 1.2] in the high volatility world.

OK, let's finish this up. The value of the L2 is independent of volatility:

(Local) In[113]:=
```
vl2dv1[q_, a_: -m`l2Stage, α_: m`α, λ_: m`λ, δ_: m`δ, amin_: -m`l2Stage,
   delay_: m`delay, dValue_: m`dValue, l3Value_: m`l3Value] :=
  ⅇ^(amin λ) l3Value q;
{vl2dv1[q, amin, α, λ, δ, amin, delay, dValue, l3Value],
  vl2dv1[q]} // Simplify
```

(Local) Out[114]=
```
{ⅇ^(amin λ) l3Value q, 0.852381 q}
```

The value of the L1 that gets to choose between L2 and L2d is

(Local) In[115]:=
```
vdv1[q_, a_: - m`l2Stage, α_: m`α, λ_: m`λ, δ_: m`δ, amin_: - m`l2Stage,
   delay_: m`delay, dValue_: m`dValue, l3Value_: m`l3Value] :=
  Max[
   vl2ddv1[q, amin, α, λ, δ, amin, delay, dValue, l3Value],
   vl2dv1[q, amin, α, λ, δ, amin, delay, dValue, l3Value]
  ];
{vdv1[q, amin, α, λ, δ, amin, delay, dValue, l3Value],
  vdv1[q] // PiecewiseExpand} // Simplify
```

(Local) Out[116]=

$$\left\{\text{Max}\left[e^{\text{amin}\,\lambda}\,\text{l3Value}\,q,\ \frac{1}{2}\left(\text{dValue}\,e^{\frac{\text{amin}\,\lambda}{\delta}}+e^{\left(\text{amin}+\text{delay}-\frac{\text{delay}}{\delta}\right)\lambda}\,\text{l3Value}\,q+\text{Max}\left[\text{dValue}\,e^{\frac{\text{amin}\,\lambda}{\delta}},\ e^{\left(\text{amin}+\text{delay}-\frac{\text{delay}}{\delta}\right)\lambda}\,\text{l3Value}\,q\right]\right)\right],\right.$$

$$\left.\begin{cases} 0.852381\,q & q \geq 1.20635 \\ 0.128146+0.746155\,q & 0.343485 < q < 1.20635 \\ 0.256293+0.373077\,q & \text{True} \end{cases}\right\}$$

(Local) In[117]:=
```
Plot[{
   e^(-(m`λ/m`δ) m`l2Stage) m`dValue, e^(-m`λ m`l2Stage) m`l3Value q,
   vdv1[q], vl2dv1[q], vl2ddv1[q]
  },
 {q, 0, 2.5},
 PlotStyle → {Gray, Gray, Directive[Thick, Black], Blue, Red},
 PlotRange → {Automatic, {0, Automatic}}
]
```

(Local) Out[117]=

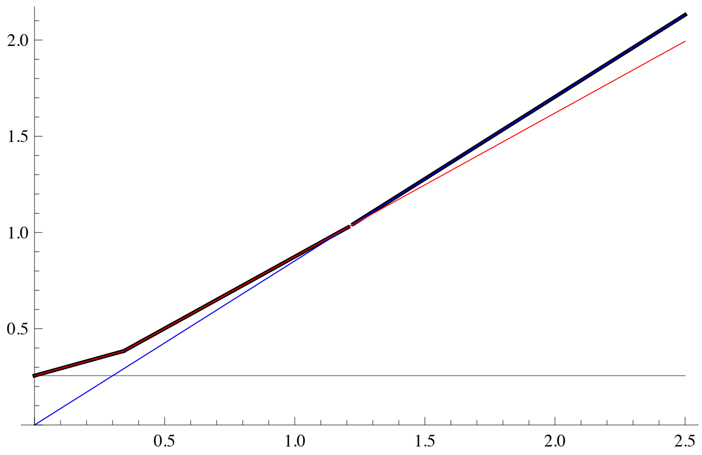

(Local) In[118]:=

```
Module[{dumbQ = 1.3, smartQ = 1.2},
 Plot[{
   vdv1[q], vsv1[q]
  },
  {q, 0, 2.5},
  PlotStyle → {{Thick, Blue, CapForm["Round"], Opacity[0.7]},
    {Thick, Red, CapForm["Round"], Opacity[0.7]}},
  PlotRange → {Automatic, {0, Automatic}},
  PlotPoints → 100,
  Frame → {{True, False}, {True, False}},
  FrameStyle → Directive[Bold, Black, FontSize → 12, FontFamily → "Helvetica"],
  FrameLabel → {"predicted environment quality", "value (dauers)"},
  Epilog → {
    Style[Text["dumb", {dumbQ, vdv1[dumbQ]}, {-1, 1}],
     Bold, Blue, FontSize → 12, FontFamily → "Helvetica"],
    Style[Text["smart", {smartQ, vdv1[smartQ]}, {1.3, -1.3}],
     Bold, Red, FontSize → 12, FontFamily → "Helvetica"]
   }
 ]
]
```

(Local) Out[118]=

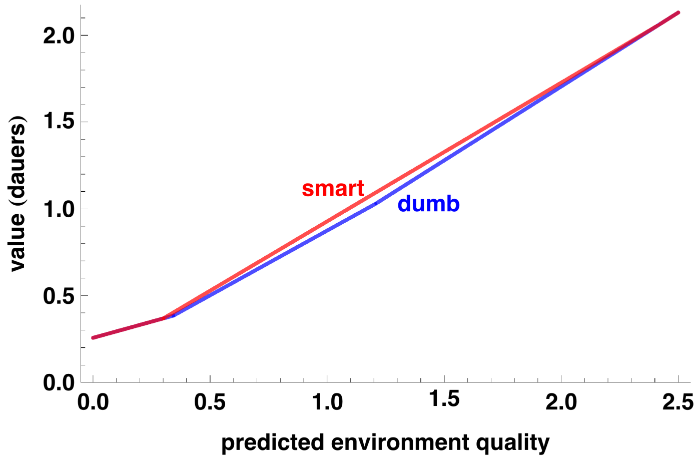

The difference between the dumb worm value and the smart worm value is:

(Local) In[119]:=
```
vdsΔv1[q_, a_: -m`l2Stage, α_: m`α, λ_: m`λ, δ_: m`δ, amin_: -m`l2Stage,
   delay_: m`delay, dValue_: m`dValue, l3Value_: m`l3Value] :=
  Evaluate[
   vsv1[q, a, α, λ, δ, amin, delay, dValue, l3Value] -
     vdv1[q, a, α, λ, δ, amin, delay, dValue, l3Value] // Simplify
   ];
{
  vdsΔv1[q, amin, α, λ, δ, amin, delay, dValue, l3Value],
  vdsΔv1[q] // PiecewiseExpand
 } // Simplify
```

(Local) Out[120]=

$$\left\{\frac{1}{2}\left(\text{Max}\left[\text{dValue}\, e^{\frac{\text{amin}\,\lambda}{\delta}},\ e^{\text{amin}\,\lambda}\,\text{l3Value}\,q\right] + \text{Max}\left[e^{\text{amin}\,\lambda}\,\text{l3Value}\,q,\ \text{dValue}\, e^{\frac{\text{amin}\,\lambda}{\delta}} + e^{\left(\text{amin}+\text{delay}-\frac{\text{delay}}{\delta}\right)\lambda}\,\text{l3Value}\,q\right] - 2\,\text{Max}\left[e^{\text{amin}\,\lambda}\,\text{l3Value}\,q,\ \frac{1}{2}\left(\text{dValue}\, e^{\frac{\text{amin}\,\lambda}{\delta}} + e^{\left(\text{amin}+\text{delay}-\frac{\text{delay}}{\delta}\right)\lambda}\,\text{l3Value}\,q + \text{Max}\left[\text{dValue}\, e^{\frac{\text{amin}\,\lambda}{\delta}},\ e^{\left(\text{amin}+\text{delay}-\frac{\text{delay}}{\delta}\right)\lambda}\,\text{l3Value}\,q\right]\right)\right]\right),\right.$$

$$\left.\begin{cases} 0. & q > 2.4127 \,||\, q \le 0.300679 \\ 0.0531133\, q & 0.343485 < q < 1.20635 \\ 0.128146 - 0.0531133\, q & 1.20635 \le q \le 2.4127 \\ -0.128146 + 0.426191\, q & \text{True} \end{cases}\right\}$$

(Local) In[121]:=
```
Plot[vdsΔv1[q],
 {q, 0, 2.5},
 PlotStyle → Directive[Thick, Black, CapForm["Round"]],
 Frame → {{True, False}, {True, False}},
 FrameStyle → Directive[Bold, Black, FontSize → 12, FontFamily → "Helvetica"],
 FrameLabel → {"predicted environment quality", "value difference (dauers)"}
]
```

(Local) Out[121]=

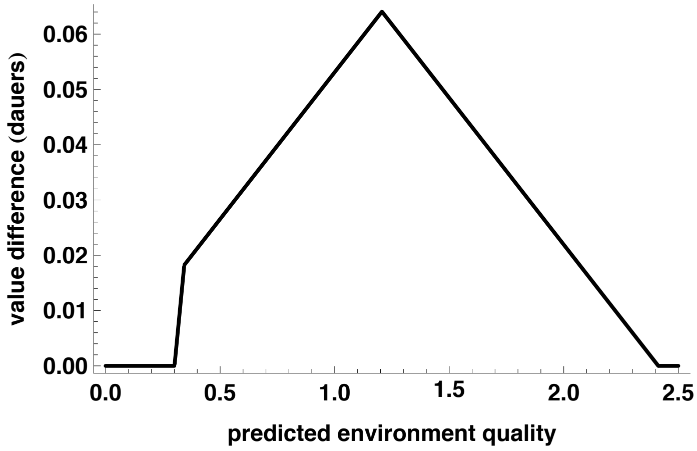

(Local) In[122]:=

```
Plot[100 vdsΔv1[q]/vsv1[q],
 {q, 0, 2.5},
 PlotStyle → Directive[Thick, Black, CapForm["Round"]],
 PlotPoints → 100,
 Frame → {{True, False}, {True, False}},
 FrameStyle → Directive[Bold, Black, FontSize → 12, FontFamily → "Helvetica"],
 FrameLabel → {"predicted environment quality", "relative value difference (%)"}
]
```

(Local) Out[122]=

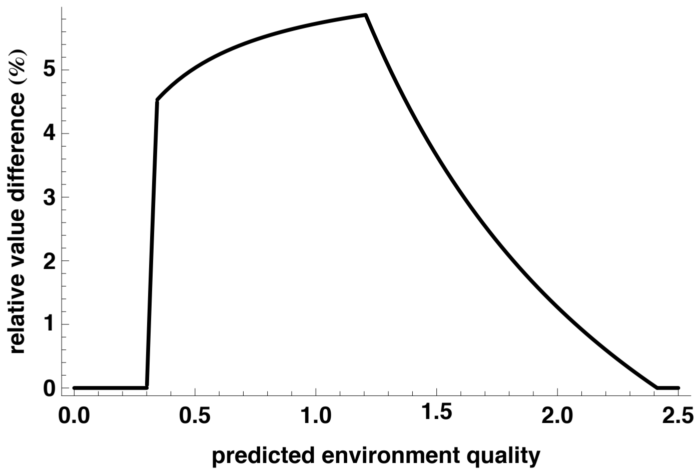

Closing

(Local) In[123]:=

```
(* End[]; *)
```
